# Multi-trait association mapping for phosphorous efficiency reveals flexible root architectures in sorghum

**DOI:** 10.1186/s12870-024-05183-5

**Published:** 2024-06-15

**Authors:** Barbara Hufnagel, Karine C. Bernardino, Marcos Malosetti, Sylvia M. Sousa, Lidianne A. Silva, Claudia Teixeira Guimaraes, Antônio Marcos Coelho, Thiago Teixeira Santos, Joao H. M. Viana, Robert E. Schaffert, Leon V. Kochian, Fred A. Eeuwijk, Jurandir V. Magalhaes

**Affiliations:** 1 Embrapa Maize and Sorghum, Sete Lagoas, Minas Gerais 35701–970 Brazil; 2 Biometris, Wageningen University and Research Center, Wageningen, 6700AC The Netherlands; 3 Embrapa Agricultura Digital, Campinas, São Paulo, 13083–886 Brazil; 4 Global Institute for Food Security, University of Saskatchewan, Saskatoon, SK S7N 4J8 Canada; 5 CIRAD, UMR AGAP Institut, Petit-Bourg, Guadeloupe F-97170 France; 6 UMR AGAP Institut, Univ Montpellier, CIRAD, INRAE, Institut Agro, Montpellier, France; 7 BASF – Nunhems, Nunhem, The Netherlands; 8 Universidade Federal do Acre, Rio Branco, Acre 69920-900 Brazil

**Keywords:** Phosphorus stress, Phosphorus acquisition efficiency, Root morphology, Root architecture, GWAS

## Abstract

**Background:**

On tropical regions, phosphorus (P) fixation onto aluminum and iron oxides in soil clays restricts P diffusion from the soil to the root surface, limiting crop yields. While increased root surface area favors P uptake under low-P availability, the relationship between the three-dimensional arrangement of the root system and P efficiency remains elusive. Here, we simultaneously assessed allelic effects of loci associated with a variety of root and P efficiency traits, in addition to grain yield under low-P availability, using multi-trait genome-wide association. We also set out to establish the relationship between root architectural traits assessed in hydroponics and in a low-P soil. Our goal was to better understand the influence of root morphology and architecture in sorghum performance under low-P availability.

**Result:**

In general, the same alleles of associated SNPs increased root and P efficiency traits including grain yield in a low-P soil. We found that sorghum P efficiency relies on pleiotropic loci affecting root traits, which enhance grain yield under low-P availability. Root systems with enhanced surface area stemming from lateral root proliferation mostly up to 40 cm soil depth are important for sorghum adaptation to low-P soils, indicating that differences in root morphology leading to enhanced P uptake occur exactly in the soil layer where P is found at the highest concentration.

**Conclusion:**

Integrated QTLs detected in different mapping populations now provide a comprehensive molecular genetic framework for P efficiency studies in sorghum. This indicated extensive conservation of P efficiency QTL across populations and emphasized the terminal portion of chromosome 3 as an important region for P efficiency in sorghum. Increases in root surface area via enhancement of lateral root development is a relevant trait for sorghum low-P soil adaptation, impacting the overall architecture of the sorghum root system. In turn, particularly concerning the critical trait for water and nutrient uptake, root surface area, root system development in deeper soil layers does not occur at the expense of shallow rooting, which may be a key reason leading to the distinctive sorghum adaptation to tropical soils with multiple abiotic stresses including low P availability and drought.

**Supplementary Information:**

The online version contains supplementary material available at 10.1186/s12870-024-05183-5.

## Background

Tropical soils are subject to intense weathering, which makes aluminum (Al) and iron oxides prevalent in the soil clay fraction. On those soils, phosphate (P) ions are rapidly and stably fixed to the Al/Fe oxides on the surface of clay minerals, making this essential macronutrient highly unavailable for uptake by plant roots. Soils with low P availability (designated henceforth as low-P soils), occupy half of the world´s agricultural lands [1] and are prevalent in the tropical world. Together, low-P availability and Al toxicity, which damages plant roots, are two important constraints for sorghum cultivation on acidic soils in sub-Saharan Africa [2, 3]. Hence, the ability of crops cultivated on low-P soils to produce grain yield per unit soil available P (i.e., P efficiency; Parentoni and de Souza Júnior [4] is of utmost importance for global food security.

The process restricting P availability on tropical soils is the movement of P from the soil clays to the root surface. In the case of tropical soils with high P fixation capacity, this movement occurs essentially via diffusion, a passive process dependent on the concentration gradient as the driving force. This passive flux of a solute in aqueous solution is described by Fick´s first law of diffusion, which has been modified by Nye and Tinker [5] and Nye [6] to describe P diffusion in the soil [7]. Once at the surface of root epidermal cells, P will be absorbed against the electrochemical gradient, with higher [P] in the root-cell symplasm and a negative inside trans-plasma membrane electrical potential, via the energy-dependent action of plasma membrane localized high- and low-affinity P transporters. Since P is found in the micromolar range in low-P soils, high affinity transporters with a low K_m_ (Michaelis Constant) are key for P uptake [8–10]. From the proposed model, P uptake by plant roots emerges as the primary factor that creates a P concentration gradient by continuously depleting P in the rhizosphere, thereby maintaining the diffusive flux of P towards the root surface. Another important factor for P diffusion on tropical soils is the soil water content [7]. In soybean, it has been shown that P diffusion on a low-P soil is severely constrained by rather small reductions in soil water [11], which makes sub-optimal P supply a rather unavoidable event on soils with high P fixation capacity, particularly for non-irrigated crops such as sorghum.

Grain yield performance under low-P has been reported to be a highly heritable trait with low genotype x environment interaction (GEI), as indicated by a high genetic correlation between control and low-P conditions, but with crossover interaction occasionally observed, particularly for high-yielding genotypes [12]. Accordingly, selection under low-P conditions was deemed adequate for sorghum improvement in West Africa. Consistent with the dynamics of P transport on low-P soils described above, the amount of rainfall was found to be the most important factor explaining GEI in low-P conditions by Leiser et al. [12]. Previously, the ability of a sorghum plant to produce grain yield under low P availability was partitioned into P internal utilization efficiency and P acquisition efficiency (PAE, Bernardino et al. [13]). These results established PAE as by far the most important component to P efficiency for sorghum cultivated in low-P soils. In addition, since grain yield assessed in low-P conditions largely reflects PAE, it is the most important trait in studies with the ultimate goal of enhancing sorghum adaptation to low P availability in the soil. The importance of P uptake traits on grain yield under low P was also reported by Leiser et al. [14].

The rationale exposed thus far clearly indicates that root system morphology and architecture traits are expected to be critical for P acquisition and hence P efficiency in tropical soils. In addition, pleiotropic QTLs and genes underlying P efficiency and root traits have already been reported [13, 15, 16]. Indeed, total root surface area and root diameter have been found to be important for grain yield under low-P availability in the soil [13, 16]. For the same amount of root biomass, a root system where finer roots prevail has higher root surface area compared to root systems dominated by thicker roots, which optimizes the ratio between active root surface area available for uptake and the root volume, which is a measure of carbon cost for root biomass formation [13, 17]. However, it is more than clear that multiple root traits, including lateral root proliferation, root hair density, root exudates, and many others are likely important for P acquisition (reviewed by López-Arredondo et al. [10]). For example, as P is mostly located in superficial soil layers, root system architecture (RSA) traits enhancing root proliferation in the surface may favor P uptake [18]. Several platforms are available for 2D and 3D root phenotyping [19–22], which generate many traits. Defining which trait or combination of traits actually lead to genetic gains is thus a significant challenge, which requires an adequate quantitative framework to be delt with [23].

Individual root morphology traits acquired with phenotyping platforms usually represent different aspects of the same biological phenomenon that ultimate define RSA. Hence, in statistical terms, these traits are typically correlated. Mixed models emerge as a useful framework to deal with multiple traits, at the same time accommodating heterogeneity in both trait correlations and variances. Mixed models have been successfully applied to multi-trait QTL analysis and led to increased detection power, at the same time helping to tease out the causes of genetic correlations between traits coming from pleiotropy or linkage [24, 25]. GWAS aimed at detecting multiple loci with minor effects using skim sequencing approaches such as genotyping-by-sequencing (GBS [26]), particularly for traits acquired in stress conditions, where heritability is in general lower compared to optimal conditions, leads to sub-optimal detection power. In turn, multi-trait genome wide association mapping (MT-GWAS) applied for dual stress related traits led to larger average effect sizes for QTLs compared to single stresses [27], which can be advantageous over single-trait GWAS.

We have previously reported on genetic resources for tackling P efficiency and root morphology traits in sorghum comprised of a large biparental, recombinant inbred line (RIL) population [13] and a multi-parent random mating population (BRP13R) [16]. We showed that these populations complement each other in terms of detection power, number of segregating alleles and, importantly, the extent of linkage disequilibrium (LD), which ultimately determines the physical resolution of a mapping approach. Here we present another component in this framework, which is built upon a multi-trait genome wide association mapping (MT-GWAS) approach in a highly diverse sorghum association panel (SAP). The framework established here, which indicates the important of flexible changes in root morphology and root system architecture potentially leading to multiple-stress adaptation in sorghum, emerges as a useful resource to finely map and validate the underlying genes.

## Results

### Characterization of single nucleotide polymorphism (SNP) markers

**Fig. 1:**
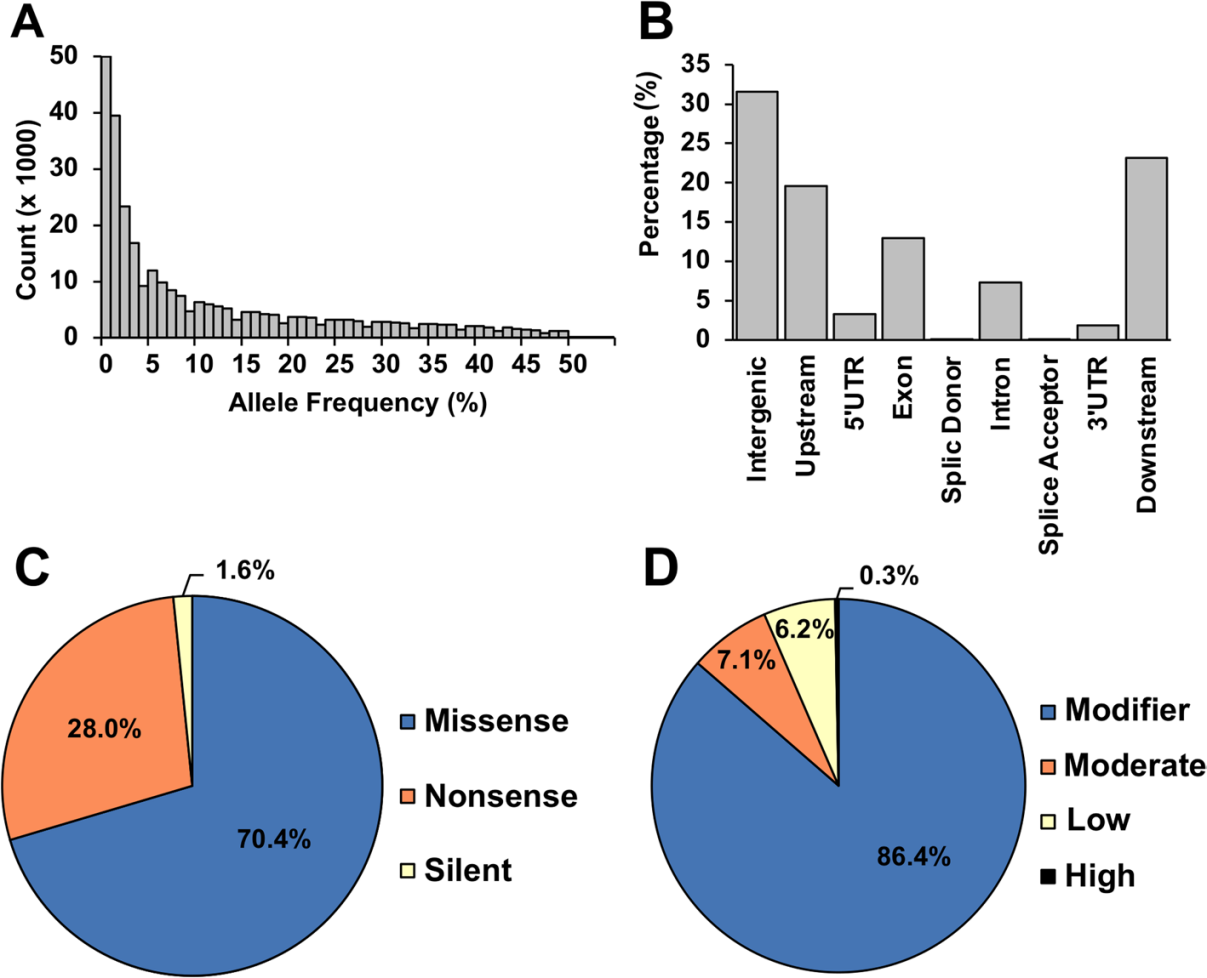
Characterization of single nucleotide polymorphism (SNP) markers obtained via genotyping-by-sequencing (GBS) for predicted effects on protein function. **A** Distribution of number of SNP loci (counts) for different classes of allele frequency. **B** Proportion of SNPs per genomic location. “Downstream” and “upstream” denote SNPs located up to 5 Kb either upstream or downstream of a gene. Proportion of SNPs in classes of nucleotide substitution (**C**) and predicted functional impact (**D**). High: SNP predicted to have a disruptive impact on the protein (e.g. generating an early stop codon, frameshift variant); Moderate, a non-disruptive variant that may change protein function (e.g. missense variant, in-frame deletion); Low, unlikely to change protein function (e.g. synonymous variant); Modifier, usually non-coding variants with no impact (e.g. downstream gene variant). A total of 295,914 (MAF > 0.01) were analyzed with the SnpEff program (Cingolani et al. [28])

A total of 295,914 SNP loci were genotyped with genotyping-by-sequencing (GBS, Elshire et al. [26]) and the average minor allele frequency (MAF) in the SAP was 0.11. Most of the marker loci had rare variants (Fig. 1A). MAF for 47% of the SNPs was under 0.05 and 37% and 16% had MAFs between 0.05 and 0.25 and exceeding 0.25, respectively. Results from a detailed analysis with the genetic variant annotation and functional effect prediction tool, SnpEff [28], indicated that the vast majority of the SNPs, 74%, were found outside genes (Fig. 1B) whereas only 13% tagged exons. Variants creating codons that produce different amino acids (i.e. missense mutations) were the most frequent substitutions, followed by 28% as nonsense mutations (Fig. 1C). Almost 90% of the variants lacked evidence for producing major functional impacts, thus possibly acting in a neutral fashion (Fig. 1D).

### Phenotypic characterization of root system morphology, architecture and P efficiency traits

We selected bi-dimensional (2D) root system morphology traits (root surface area and root diameter) and three-dimensional (3D) root system architecture traits (centroid, median roots and convex hull, Clark et al. [19]) to phenotype in a low-P hydroponics system (Table S1). For root traits, selection was done considering trait correlations (only one trait was kept when their correlation was equal or higher than 0.85) to avoid redundancies, as well as inspection of the residual variances (after log transformation, when needed) and the biological significance of each trait on P efficiency (see rationale in the discussion session). P efficiency was assessed based on sorghum performance traits under low-P conditions in nutrient solution (shoot dry weight and shoot P content) and grain yield on a low-P soil in the field.

**Fig. 2:**
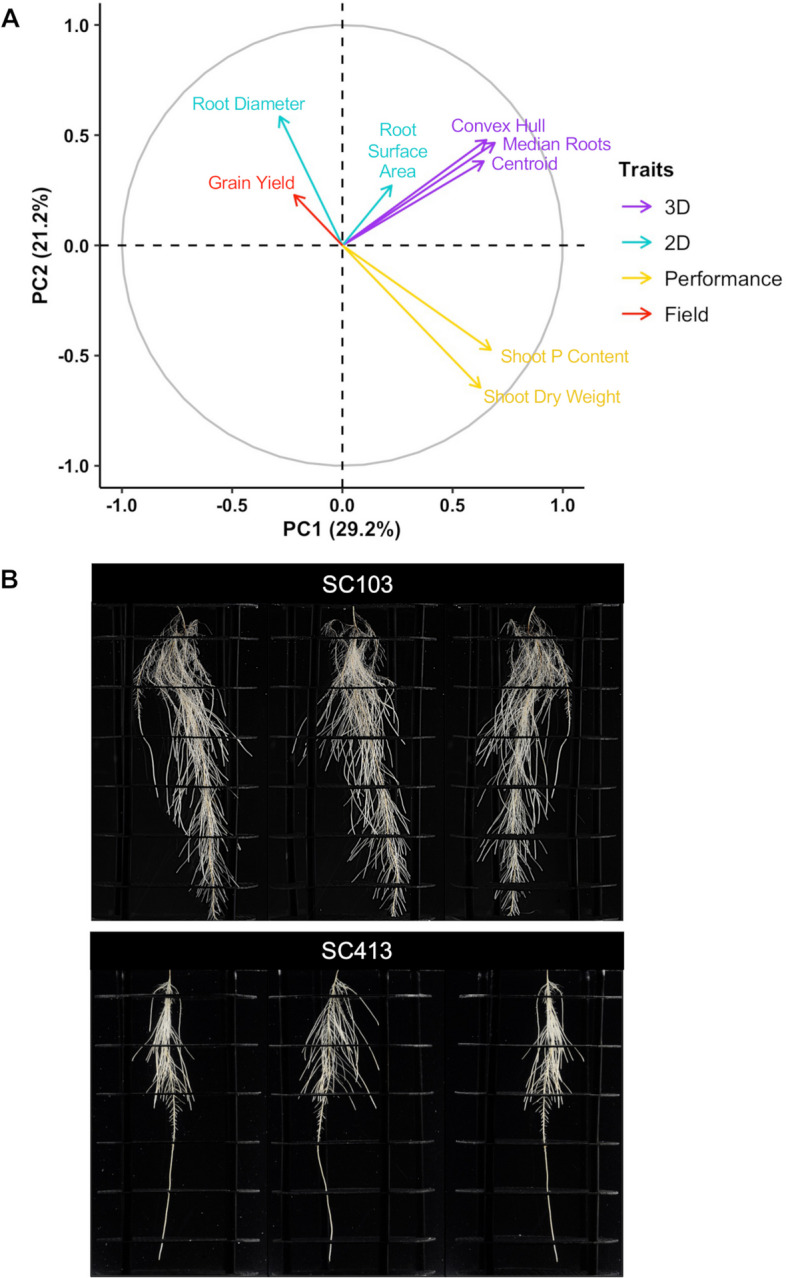
Principal component analysis (PCA) for eight traits selected for the multi-trait sorghum association mapping and root images for the SAP lines, SC103 and SC413, which are presented as examples of lines from the SAP that contrast for root system architecture traits. **A** Traits utilized for the PCA were assessed in low-P conditions in hydroponics and in the field. 2D and 3D refer to root morphology and root architecture traits, respectively. The root morphology traits are root surface area (SA) and root diameter (RD), while centroid (Cent), median roots (MedR) and convex hull (CH) describe architectural traits. We also measured the performance traits in low-P hydroponics, shoot dry weight (SDW) and shoot phosphorus content (SPCnt). Grain yield (GY) determined for sorghum lines grown on a low-P soil in the field was included to reflect P efficiency. **B** Root images for the lines SC103 and SC413, which contrast for centroid, convex hull, median number of roots and root surface area, with SC103 showing higher values for all of these traits

Heritability estimates for root morphology and architecture traits were ~ 0.8 and 0.4–0.6, respectively (Table S1). High heritability was also observed for performance traits including grain yield under low-P, while heritability for shoot P content was ~ 0.3. The three root architecture traits were positively correlated (*r* ~ 0.4–0.6, *p* ≤ 0.01), whereas a small positive correlation was observed between centroid and root surface area (*r* ~ 0.16, *p* = 0.02, Table S2 and Fig. 2A). The sorghum lines in Fig. 2B were selected based on contrasting centroid with SC103 showing higher centroid compared to SC413. In agreement with the trait correlations, compared to SC413, SC103 also showed much higher values of median number of roots and convex hull, in addition to higher total root surface area.

The performance traits assessed in hydroponics, shoot dry weight and shoot P content, were highly correlated (*r* = 0.72, *p* ≤ 0.01) and shoot dry weight was slightly and negatively (*r* = -0.16, *p* = 0.02) associated with grain yield (Table S2). Particularly, shoot P content was correlated with all root morphology and architecture traits, in general in a positive way, except for a negative correlation with root diameter, which was the same general trend observed for shoot dry weight. Except for root surface area, which was positively correlated with grain yield assessed in a low-P soil (*r* = 0.21, *p* ≤ 0.01), Table S2), and to a lesser extent, root diameter (*r* = 0.12, *p* = 0.08), no significant correlations between individual root traits and grain yield were observed. Principal components (PC) 1 and 2 explained a substantial amount of the total variance (Fig. 2A), which were more highly influenced by root architecture traits and performance traits assessed in hydroponics (i.e. these traits were highly correlated with the components as indicated by the projections of each vector, (trait) to the axes in Fig. 2A).

### Multi-trait GWAS

#### Marker-trait associations

**Fig. 3:**
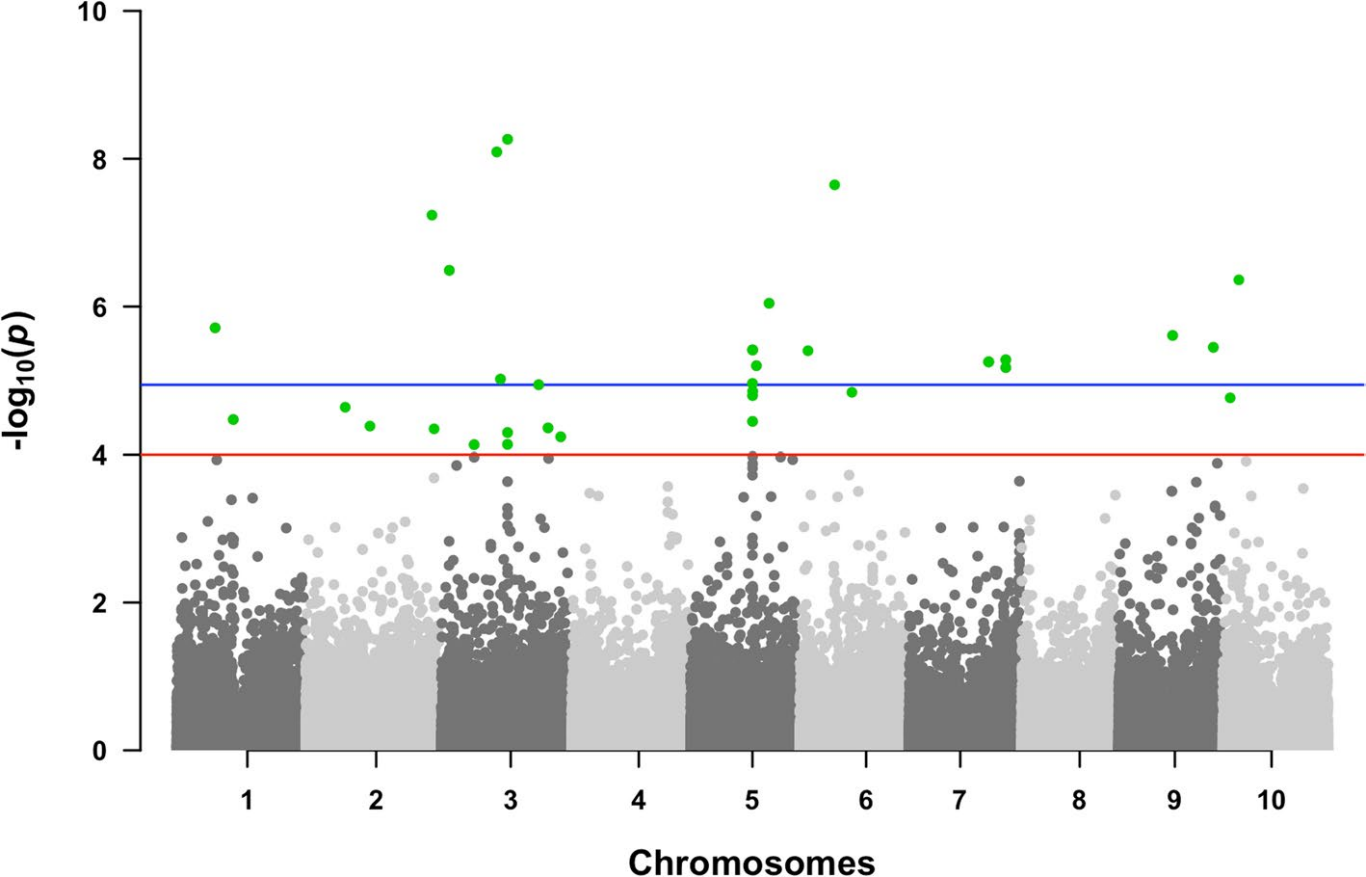
Manhattan plot for multi-trait genome-wide association mapping (MT-GWAS) in the sorghum SAP panel. Traits were assessed in low-P conditions in hydroponics and in the field on a low-P soil. The root morphology traits are root surface area and root diameter, while centroid, median number of roots and convex hull describe architectural traits. Also included are the performance traits, shoot dry weight and shoot phosphorus content acquired in a low-P nutrient solution, and grain yield assessed in a low-P soil. The blue line represents a genome-wide significance threshold (-log_10_(*p*) = 4.94), calculated with a Bonferroni correction (Blant and Altman, 1995). *Alpha* = 0.05 and the number of independent tests was calculated based on an average extent of LD of 150 Kb (Morris et al. [29]). The red line represents a -log_10_(*p*) = 4.0 threshold used to identify suggestive associations (4.0 < -log_10_(*p*) < 4.94). Statistically significant SNPs (above the blue threshold line, -log_10_(*p*) ≥ 4.94) and suggestively significant SNPs (between red and blue lines) are highlighted in green

Only SNPs with MAF > 0.025 were used for GWAS for a total of approximately 195,656 marker/trait association tests. Based on the Akaike information criterion (AIC), the factor analytic order 2 model (FA2) was selected for the covariance matrix between traits. A total of 24 SNPs distributed on all sorghum chromosomes, except for chromosomes 4 and 8, were found to be significantly associated with the multi-traits, and another 11 SNPs were considered suggestive for trait associations (4 ≤ -log_10_(*p*) < 5, Fig. 3). The physical positions and association signals for these SNPs are in Table S3. Five markers, S3_19022691, S5_35888696, S5_36013572, S7_56277752 and S7_56277754 showed pleiotropic effects for grain yield, root morphology and P-efficiency. The strongest associations (-log_10_(*p*) = 7.9–8.8) were found for the SNP loci S3_32105315 and S3_38375664, which are 6.27 Mb apart on chromosomes 3, and for S6_20279639 on chromosome 6 (Table S3). Other strong associations (-log_10_(*p*) > 6) were found for SNPs on chromosomes 1 (S1_22277047), 2 (S2_73237482), 3 (S3_4627500), 5 (S5_35992354, S5_35992361 and S5_45530884), 7 (S7_46348538 and S7_ 46,348,542), 9 (S9_31332114) and 10 (S10_9309043).

Linkage disequilibrium based on the squared genotypic correlations between pairs of loci (r^2^) [30] was estimated for all associated SNP loci physically linked on the same chromosome (Table S4). As expected, SNPs separated by only a few base-pairs on chromosomes 5 and 7 were in strong LD (r^2^ > 0.9, *p*-value < 1.94 × 10^−18^). SNP loci apart by larger distances were not found to be under strong LD and may tag causative loci acting independently on root and/or P efficiency traits, although some degree of long-range LD possibly occurs (e.g. S3_38375664 vs. S3_56401131).

### SNP effects

The proportion of the phenotypic variance for grain yield explained by associated SNPs was small, ranging from less than 1–2% (Table S3). For grain yield, S3_19022691, S5_35888696, S5_36013572, S7_56277752, S7_56277754 explained ~ 2% of the phenotypic variance. The largest proportion of the phenotypic variance was explained by SNP loci associated with root surface area and shoot dry weight. SNPs associated with root surface area explained 7.5% (± 2.8%) of this trait variation in average and a maximum value of almost 20% was found for S3_38375664. SNPs associated with shoot dry weight in low-P explained ~ 7.0% (± 3.5%) of the phenotypic variation in this trait. The SNPs that explained a large proportion of the root surface area phenotypic variation, S3_32105315, S3_38375664 and S6_20279639, were also above average for variation explained for traits other than grain yield. The SNP, S3_32105315, also explained a comparatively large proportion of the phenotypic variation for grain yield, of ~ 1% compared to an average of 0.5% across all associated loci.

**Fig. 4:**
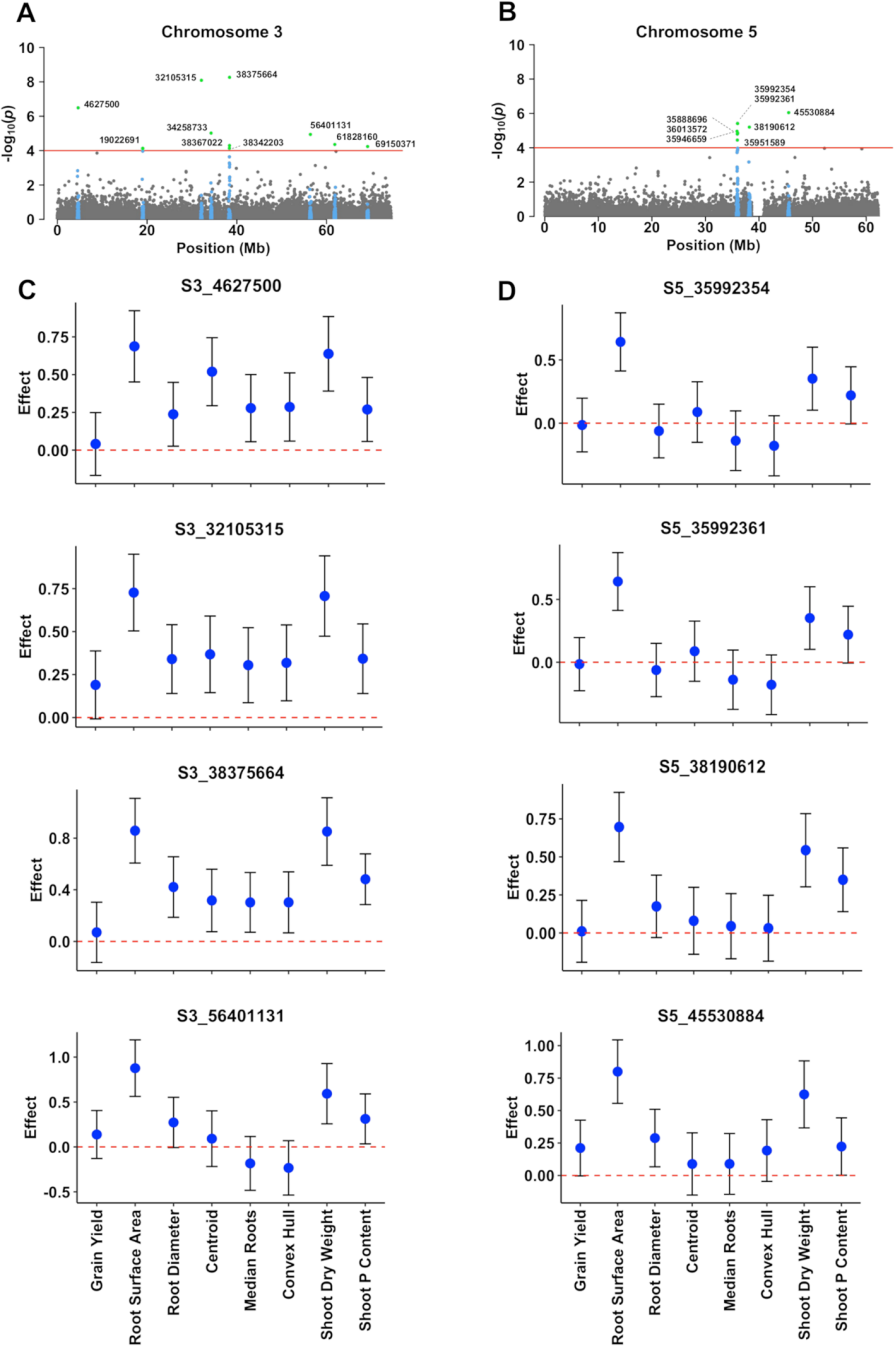
Estimated effects for SNPs that were significantly associated with multiple traits by MT-GWAS. The physical coordinates (in base pairs, bp, based on the sorghum genome version 2.1), next to each associated SNP are shown for SNPs on chromosomes 3 (**A**) and 5 (**B**). Associated SNPs (green) and SNPs within a physical window of 150 Kb (in blue, depicting the estimated extent of LD in sorghum, Morris et al. [29]), around the associated SNPs are highlighted. The red dashed line depicts the -log_10_(*p*) = 4.0 threshold. Estimated effects for SNPs on chromosomes 3 and 5 with maximum -log_10_(*p*) by MT-GWAS are shown in (**C**) and (**D**), respectively (explained phenotypic variances for each SNP are in Table S3). The SNP designations shown in (**C**) and (**D**) consist of the letter “S” (SNP) followed by the respective chromosome number and physical position in bp. Estimated SNP effects (blue dots) and 95% confidence intervals (vertical line) are shown. The horizontal red dashed line at zero indicates there was no statistically significant difference for the effect of the two homozygous classes at each SNP locus. Hence, the confidence intervals for significant SNPs do not overlap with the dashed line. The effect signs, either positive or negative, indicate the origin of the allele that increases phenotypic expression of a given trait (i.e. favorable allele). SNPs with positive effect signs have the minor allele (allele with a frequency < 0.5) as favorable, whereas negative signs indicate that the alleles with frequency > 0.5 (major allele) increase the phenotype. All traits were standardized before MT-GWAS to have zero means and total phenotypic variance equal to 1

In the cases where significant correlations between traits were detected, those correlations were in general positive (Table S2). Consistent with that, in general, the alleles at associated SNP loci that increased phenotypic expression (i.e. favorable alleles) were the same for the different traits. Notable rare exceptions were for the root 3D traits, median roots and convex hull (S3_56401131, S5_35992354 and S5_35992361) (Fig. 4). We observed several instances where, for each of the traits, the confidence intervals for most of the SNPs do not extensively overlap with zero (Fig. 4 and Figs. S1 – S5). Such cases where the same alleles consistently increase phenotypic expression across most of the root system morphology and architecture traits and the P efficiency traits, including grain yield in low-P conditions, were observed for markers S3_32105315 and S5_45530884 (Fig. 4), S2_73237482 (Fig. S1), S3_19022691, S3_34258733, S3_38367022, S3_61828160 and S3_69150371 (Fig. S2), S5_35888696, S5_35951589 and S5_36013572 (Fig. S3), S6_4827069, S6_20279639, S6_30277605, S7_46348538, S7_46348542, S7_56277752 and S7_56277754 (Fig. S4), S10_9309043 (Fig. S5). Examples where the minor allele was favorable (20 SNPs) were much more common compared to the major alleles (4 SNPs) (Table S3).

### QTL conservation across mapping populations

**Fig. 5:**
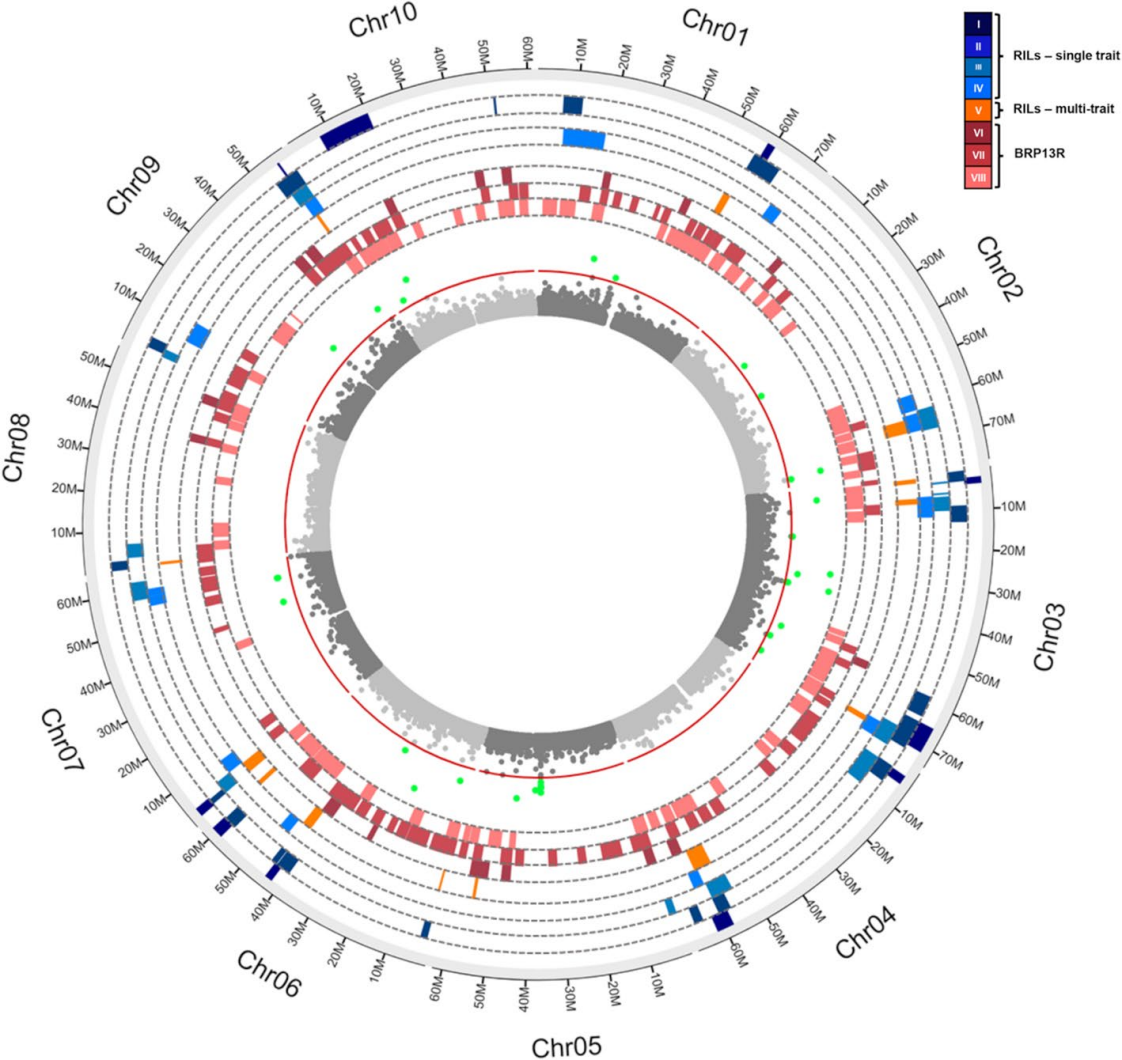
Circos plots for QTLs detected in the RIL population (Bernardino et al. [13]), on the BRP13R population (Bernardino et al. [16]), and on the sorghum association panel (present study). Field traits: grain yield (GY), flowering time (FT), plant height (PH), grain dry matter (GDM), plant dry matter (PMD), plant phosphorus content (Pp), grain phosphorus content (Pg), total phosphorus content (Pt). Root morphology traits (nutrient solution): root diameter (RD), root length (RL), root surface area (SA), surface area of superfine roots (SA1, 0 mm < RD ≤ 1 mm), surface area of fine roots (SA2, 1 mm < RD ≤ 2 mm), surface area of thicker roots (SA3, 2 mm < RD ≤ 4.5 mm), root volume (RV), volume of fine roots (V2, 1 mm < RD ≤ 2 mm), relative net root growth (RNRG, Al tolerance measure). Seedlings sorghum performance traits and P uptake (low-P nutrient solution): shoot dry matter (SDM), root dry matter (RDM), shoot phosphorus content (Ps), root phosphorus content (Pr). The positions of QTLs detected in the RILs by single- (blue rectangles) and multi-trait mapping (orange), and of QTLs detected in BRP13R by GWAS (red), are shown. The eight concentric circular tracks composed of colored rectangles (from the outer to the inner position, as represented in the schematics on the right), depict the positions of QTLs. QTLs from the single-trait mapping in RILs: (I) GY, (II) FT, PH, Pp, Pg, (III) RD, SA, SA2, SA3, RV, V2, (IV) SDM, RDM, Ps, Pr. (V) QTLs from the multi-trait mapping in RILs. QTLs from GWAS in BRP13R: (VI) GY, (VII) PDM, GDM, Pp, Pg, Pt, FT, PH, and (VIII) RNRG, RL, RD, SA, SA1, SA2, SA3, RV, V2, SDM, RDM, Ps, Pr. MT-GWAS results in the SAP are in the inner circle. Associated SNPs are in green; the red circular line represents a -log_10_(*p*) = 4 threshold

Next, we looked at conservation between the regions detected by MT-GWAS in the sorghum association panel in the present study and quantitative trait loci (QTLs) underlying P efficiency and root traits in a large RIL population [13] and a multi-parent random mating population [16]. These QTLs are graphically depicted in Fig. 5 and detailed physical information is shown in Table S5. About 80% of the 24 regions where significant SNPs were found by MT-GWAS in the SAP co-located with QTLs previously detected in other populations phenotyped for similar traits assessed in low-P conditions. Hence, only six regions appear to be completely specific to the SAP.

As depicted in detail in Table S5, instances of regions detected by MT-GWAS in the SAP co-locating with QTL for the performance traits, grain yield or grain dry matter assessed in low-P conditions were found on chromosomes 1 (20–25 Mb), 2 (70–75 Mb), 3 (0–5 Mb, 55–60 Mb, 65–70 Mb), 6 (0–5 Mb, 25–30 Mb), 9 (50–55 Mb) and 10 (0–10 Mb). In addition, regions on chromosomes 1 (30–35 Mb), 3 (60–65 Mb), 5 (50–55 Mb), 6 (15–20 Mb), 7 (45–50 Mb, 55–60 Mb) and 10 (0–5 Mb) were conserved with regards to QTLs controlling biomass accumulation and/or P uptake in low-P conditions.

A total of nine QTLs detected by MT-GWAS in the SAP co-located with root morphology QTL, particularly root surface area (SA) and root diameter (RD) and, to a lesser extent, root volume (RV) (Table S5). Those are located on chromosome 1 (20–25 Mb, RD), 2 (20–25 Mb, SA), 3 (0–5 Mb, RD, 65–70, SA and RV), 5 (50–55 Mb, RV), 9 (50–55 Mb, RD) and 10 (0–5 Mb, 5–10 Mb, SA and RV). Therefore, instances of root morphology QTL conserved with regards to performance traits under low-P were observed. Those conserved QTL are located on chromosomes 1 (20–25 Mb), 3 (0–5 Mb, 55–60 Mb, 60–65 Mb, 65–70 Mb), 5 (50–55 Mb), 7 (55–60 Mb), 9 (50–55 Mb) and 10 (0–5 Mb).

### Relationship between root system architecture and morphology traits assessed in hydroponics and in a low-P soil

We evaluated root architecture and morphology and sorghum grain yield in a low-P soil in order to establish the connection with root traits assessed in hydroponics in the SAP. For that, four sorghum genotypes contrasting for centroid assessed in hydroponics with high (SC25 and SC103) and low (SC1080 and SC413) centroids were cultivated in cylinders filled with a high P-fixing Oxisol (Latossolo Vermelho Distrófrico, Santos et al. [31]) with a clayey texture (64% of clay), under savannah vegetation (see Fig. 2 for the contrasting root systems of SC103 and SC413, with centroids of 9.08 and 4.82). The maximum and minimum values of the center of mass of the root system (i.e. centroid) in the SAP based on hydroponics imaging were 9.08 and 0.99 cm, respectively, with a mean of 5.44 ± 1.4. Hence, SC25 (centroid 8.92 cm) and SC103 (9.08) can be considered genotypes with high centroid whereas SC1080 (2.62 cm) and SC413 (4.82) have low centroids.

**Fig. 6:**
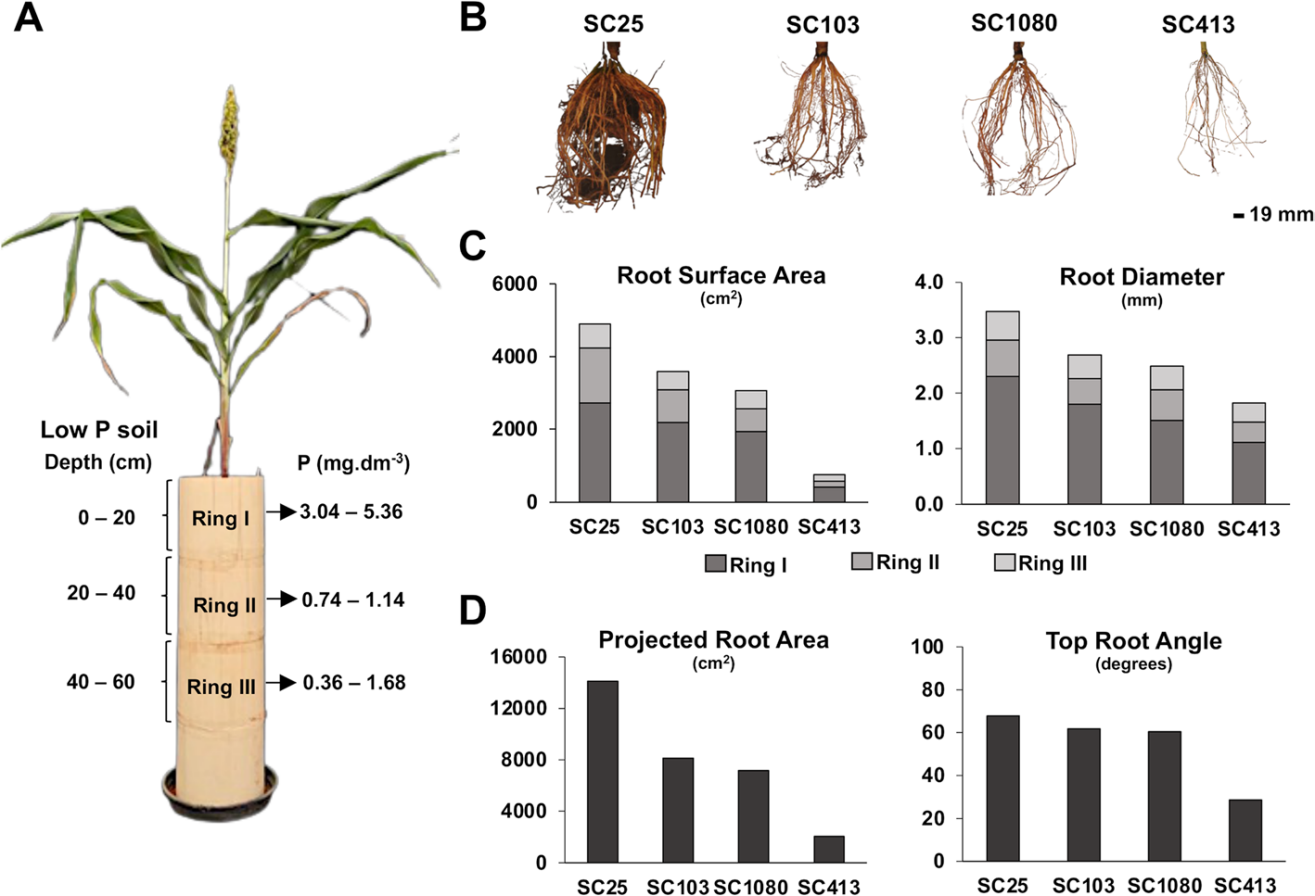
Assessment of RSA in a low-P soil. **A** Schematic of the cylinder system used for root phenotyping in the soil. Three layers of soil from a high P fixing Oxisol (low-P soil, Latossolo Vermelho Distrófrico, Santos et al. [31]) with 64% clay were used to fill PCV cylinders. The 0–20 and 20–40 cm soil layers were used to fill Rings I and II, respectively, while Ring III and the ring underneath it were filled with the 40–80 cm subsoil layer. Available P (mg.dm^-3^ Mehlich1 P) in the original soil is shown. **B** Images of the root system of the sorghum genotypes contrasting for high (SC25 and SC103) and low (SC1080 and SC413) centroid assessed in hydroponics. **C** Root morphology analysis: Root surface area (SA, cm^2^) and root diameter (RD, mm). **D** Root architecture analysis: projected root area (cm^2^) and top root angle (degrees). Statistical analysis is shown in Tables S6 and S7

We set up a growing system (Fig. 6A) where the top ring was filled with the topsoil (Ring I, 0–20 cm soil layer) whereas Rings 2 and 3 were filled with soil from sub-superficial layers (20–40 cm and 40–80 cm, respectively). The soil P availability in the original soil was extremely low, between 0.1 and 1.0 mg dm^−3^ (Mehlich1 extractor). The rings were bound together using plastic adhesive tape, which allowed us to excise the first three rings and assess RSA and root morphology traits at increasing depths up to 60 cm in the soil profile upon harvest.

Root image analysis shows that SC25 and SC103 have more developed root systems than SC1080 and SC413, with SC25 and SC413 greatly contrasting for high and low root system development, respectively (Fig. 6B). We observed significant genotypic differences in root morphology, namely surface area and diameter, in addition to a significant interaction between genotypes and soil depth (Table S6) for both traits. Comparing root morphology traits of sorghum genotypes within each soil depth (Fig. 6C and Table S6), those differences were found to be in general consistent with the more extensive root systems of genotypes with higher centroid, that is, higher root surface area in SC25 and SC103 compared to SC1080 and SC413, particularly up to 40 cm soil depth. The differences in surface area between genotypes reduced as the depth of the root system increased, almost disappearing between 40 and 60 cm, except for the much less developed root system of SC413 (Fig. 6C and Table S6). In addition, although with much smaller differences, SC25 and SC103 tended to show slightly thicker roots compared to SC1080 and SC413 (Fig. 6C and Table S6), particularly in the most superficial soil layer.

Root system architecture analysis with the Digital Imaging of Root Traits (DIRT [32], software (see modification in the Materials and Methods) indicated that the projected root area was the highest in SC25, similar in SC103 and SC1080 and much lower in SC413 (Fig. 6D, Table S7). The median width of the root system was higher in SC25 and SC1080 than in SC103/SC413 and SC413 showed a much smaller top root angle compared to the other genotypes. Phosphorus efficiency measured as grain yield in a low-P soil was highest in SC25 and by far the lowest in SC413, whereas SC103 and SC1080 showed intermediate P efficiency (Table S7).

## Discussion

By exploring historical recombination events, genome wide association mapping (GWAS) can be used for finely mapping genes underlying important agricultural traits such as P efficiency in low-P soils. Based on our analysis with the genetic variant annotation and functional effect prediction tool, SnpEff, the associations found by GWAS at the marker density provided by GBS are primarily caused by indirect associations from proxy SNPs in LD with unobserved causative loci, which is in line with insufficient marker saturation in GWAS approaches in sorghum [33]. Particularly with regards to complex traits, GWAS in highly diverse association panels such as the SAP [34] is severely limited by the effect and frequency of the causative mutations [35, 36] which, in a context of low LD, are not efficiently tagged by marker systems based on shallow sequencing approaches [13]. Despite these limitations, more sophisticated strategies such as multi-trait GWAS, particularly when integrated with marker-trait association approaches in populations with complementary genetic properties, can greatly contribute to the understanding of the causes of correlation between traits and also increase detection power [24, 25, 27], thus contributing to better mechanistic understanding of complex traits.

We assessed via MT-GWAS those traits that have been previously implicated in sorghum P efficiency, to elucidate the role of root system morphology and architecture in this important agronomic trait for tropical agriculture [2, 3]. Hence, we selected the root morphology traits, root diameter and root surface area, which we previously showed were associated with P efficiency QTL in sorghum [13, 16]. Topsoil foraging has been deemed to be an important adaptation to enhance P acquisition in low-P conditions in beans [37]. In sorghum, we previously saw that root architecture traits such as centroid, which measures the tendency of a root to grow and produce significant root biomass at different depths, were associated with allelic variation for *SbPSTOL1* genes that were found to enhance P efficiency in sorghum [15]. Hence, we included in this analysis the root architecture traits, centroid, median roots (i.e. median number of roots from root counts taken from all horizontal cross-sectional slices through the entire root system) and convex hull (i.e. volume of the convex enclosure that encompasses the whole root system; Clark et al. [19]). Finally, in order to get insights into the role of root architecture on P efficiency, we assessed traits reflecting sorghum performance under low-P in hydroponics, namely shoot dry weight and shoot P content, as well as grain yield assessed on a low-P soil, in the field.

In particular, the impact of changes in root system architecture such as those enhancing root proliferation in the soil surface horizon, where P is generally at a higher concentration, remains hard to identify in studies on sorghum P efficiency. While nodal root angle, which influences root distribution in the soil profile, has been associated with drought tolerance [38, 39], Marcus [40] questioned its role in sorghum adaptation to P deficient soils, at least at an early stage of sorghum development. In contrast, Parra-Londono et al. [41] indicated that sorghum adaptation to low-P involves root system architectures that benefit topsoil foraging, such as a bushy and shallow root systems.

Our results with MT-GWAS indicated that the same alleles of SNPs associated with multiple traits measured under low P conditions, including grain yield, increased phenotypic expression of the different traits. Such cases are, for example, for the chromosome 3 SNPs, S3_19022691 (Fig. S2), S5_35888696 on chromosome 5 (Fig. S3), S7_562777452 on chromosome 7 (Fig. S4), among others. The RSA trait, centroid, is defined as the vertical position of the center of mass of the entire root system relative to the seed, with a larger centroid value being indicative of a more deeply growing root system [19]. Accordingly, in rice, the root system of Azucena, with its large centroid value, tends to grow deeper with less root biomass and branching near the top of the root system (see Fig. 5 in Clark et al. [19], for the root phenotypes of Azucena and IR64). In sorghum, we observed that genotypes with a larger centroid have root types that are more branched not only at depth within the soil, but also throughout the entire root system, in addition to having a deeper root system (Fig. 2B). We found that the three RSA traits, centroid, median roots and convex hull were positively correlated (*r* = 0.4–0.6, *p* ≤ 0.01) and centroid was also positively correlated with root surface area, although not as strongly (Table S2 and Fig. 2A). These findings suggest that those traits have a unified developmental origin, at least in part. Root systems with a larger median root number and convex hull are expected to have a more branched root system that is able to access a larger soil volume [19, 42], being thus able to acquire P more efficiently.

Our analysis of root system architecture in a low-P soil (Fig. 6B) indicates a general agreement with centroid assessed in hydroponics (see SC103 vs. SC413 in Figs. 2B and 6B), with centroid (i.e. vertical position of center of mass of the entire root system, Clark et al. [19]) largely reflecting overall root development. P efficiency measured via grain yield under low P was found to be consistent with higher root surface area, particularly up to 40 cm, with genotypic differences in surface area disappearing in the deeper soil layer between 40 and 60 cm. Hence, these results indicate that genotypic differences in root surface area compatible with P efficiency occur exactly in the soil layers where P is at the highest concentration, suggesting a specific mechanistic response to enhance P nutrition under low P availability in the soil. Although the differences in root surface area clearly reduced as the soil depth increased, even at 40–60 cm, there was no indication that higher root development in the surface, such as for SC25, comes at the expense of deep rooting, at least when the most important factor for nutrient and water uptake, surface area, is taken into consideration. Our results also indicate that centroid is highly influenced by the root mass distribution in the soil profile and can be better described by multiple traits such as the combination of root angle and surface area, particularly in shallower soil layers. The closer relationship between grain yield and root surface area compared to root angle suggests that changes in root surface area are the primary factor leading to changes in root system architecture traits such as centroid, leading to genotypic differences enhancing P diffusion from the soil to the root surface, P uptake and, finally, grain yield under low P availability in the soil. This is reinforced by the results in the sorghum association panel, given that root surface area was the only root trait correlated with grain yield in a field site with low P availability. We also argue that a proper description of root phenotypes related to P efficiency is essentially multivariate in nature, which justifies our multi-trait GWAS approach to study this problem. A root ideotype has been proposed to optimize both water and N acquisition in maize [43]. One common aspect of the proposed ideotype and our findings is the importance of enhanced lateral root proliferation, which shall ultimately define the total surface area of a crop root system. This trait may benefit not only P efficiency, as shown here in sorghum and in maize by Zhu and Lynch [44], but also possibly favoring acquisition of more mobile elements, such as N.

Hence, our MT-GWAS results support the contention that a deeper root system in sorghum does not necessarily occur at the expense of root branching in the soil surface, and that sorghum genotypes are flexible enough to accommodate both traits. This may in part be controlled by pleiotropic loci as indicated by our MT-GWAS results, leading to a higher P uptake and enhanced grain yield in low-P soils. A previous study indicated the importance of pleiotropic loci with a significant impact in known dwarfing genes and on root architecture [33]. This result is significant as crop adaptation to acidic soils is the result of adaptation to multiple stress factors, as the joint outcome of Al tolerance, P efficiency and drought tolerance. In rice, *DEEPER ROOTING 1 (DRO1)* controls deep rooting, conferring drought avoidance [45]. Our MT-GWAS results suggest that genes controlling deep rooting in sorghum may pleiotropically lead to a more branched root system both in the surface and in the subsoil, which could benefit overall sorghum adaptation to acidic soil environments that are impacted both by low-P availability and drought stress. This lack of conflict from the root architecture perspective between P efficiency and water acquisition may be a key factor leading to the inherent ability of sorghum to withstand abiotic stresses in harsh environments, allowing the sorghum crop to show yield stability under significant levels of abiotic stress.

We have previously reported on a genetic framework for the identification of genomic regions controlling P efficiency and root system morphology and architecture in sorghum, based on genetic mapping using multiple populations with complementary genetic properties, such as the integrated use of recombinant inbred lines [13] and a multi-parent random mating populations [16]. Here, we integrated MT-GWAS using the SAP in this framework (Fig. 5 and Table S5) to finely map P efficiency loci and to help in the identification of the underlying genetic determinants, taking advantage of the comparatively low LD in this highly diverse sorghum panel. Interestingly, we found that sorghum chromosome 3 is a hub for QTLs underlying root traits and P efficiency, which was similar to the findings reported by Parra-Londono et al. [41].

The genetic properties inherent to each population in our P efficiency mapping framework leads to the detection of population-specific QTLs, such as strictly overdominant QTL for grain yield that were detected in the BRP13R random mating population, taking advantage of this population’s residual heterozygosity [16]. However, conserved QTL across populations that were frequently observed can also be used to identify candidate genes for further validation approaches.

For example, we found several conserved QTL in the end region of chromosome 2 at ~ 73 Mb, and a serine-threonine kinase gene, Sobic.002G375500, was found approximately 50 Kb from SNP S2_73237482, which produced a strong association signal with the multi-traits. Both in rice and in sorghum, the serine-threonine kinase genes, *PSTOL1*, were found to modulate root morphology and P efficiency [15, 46].

Another very interesting region is the end region of sorghum chromosome 3, where QTLs underlying yield in low-P conditions were mapped between 60 and 75 Mb (Table S5) in our RIL population [13] and in the BRP13R population [16]. We found in the SAP that the SNP, S3_69150371 is associated with the multi-traits (Fig. S2), narrowing down this putative conserved QTL region to the 65–70 Mb interval on chromosome 3. Within this region lies Sobic.003G37790, which is similar to the Arabidopsis transcription factor, *SCARECROW* (*SCR*) [47]. SCR has been implicated in asymmetric cell division in roots [48, 49]. In Arabidopsis, the *scr-1* T-DNA mutation was identified on the basis of a dramatic reduction in root length in seedlings [50]. In white lupin, SCR homologs have been implicated in lateral root development, which may contribute to lupine adaptation to low-P soils [51–53]. The effect of SCR on root development originates very early in the developmental process, during embryogenesis [50], which could explain the strong pleiotropic effect on RSA, root morphology and P efficiency captured by S3_69150371. On chromosome 9, S9_54933392 is associated with the multi-traits and overlaps with grain yield QTLs in the other mapping populations. Only ~ 5 Kb from this SNP marker lies a sorghum gene similar to lateral organ boundaries (LOB) genes. The LOB genes encode transcription factors that are important elements in auxin signal transduction [54], influencing root development and root architecture plasticity [54, 55]. They also have been implicated in lateral root formation (reviewed by [51]) and have been shown to be modulated by P status in maize [56].

## Conclusions

The present study indicates that enhanced lateral root development is an important trait for sorghum adaptation to low-P soils and is not contingent on shallow rooting, particularly concerning root surface area, which is pivotal for water and nutrient uptake. Hence, this response could also be important for coping with the multiple abiotic soil stresses of P deficiency, Al toxicity and drought, which are often co-occurring for crops cultivated on acidic tropical soils. In addition, the molecular genetic framework detailed here, which comprises QTLs and GWAS peaks for loci associated with P efficiency and root traits detected in different populations, highlights the end region of sorghum chromosome 3 as important for P efficiency. This integrated resource can now be used to identify the underlying determinants of abiotic stress tolerance in sorghum as a prelude to functional validation via gene editing and other approaches. The resulting genes and regulatory factors can be the basis of molecular breeding and biotechnological approaches integrating important root architecture traits, which can foster the development of sorghum cultivars with enhanced yield stability for cultivation on acidic soils that are abundant in tropical regions.

## Materials and methods

### Genetic material

This study was performed with 272 sorghum lines that are a subset of the sorghum association panel (SAPst, Hufnagel et al. [15]) described by Casa et al. [34]. This panel consists of tropical converted and breeding lines chosen to represent the genetic diversity of cultivated sorghum.

### Phenotyping

#### Field experiments

Procedures for assessing grain yield (GY, kg ha^−1^) in low-P conditions were previously reported by [15] in a study on the role of sorghum homologs of *Phosphorus Starvation Tolerance1* (*PSTOL1*) genes on P efficiency. Briefly, a total of 243 3-dwarf lines from the SAP were evaluated for P efficiency-related traits in a site with low-P availability in 2011. Experiments were set up as three lattice designs with nine incomplete blocks, nine accessions from the SAP and two checks (ATF13 and ATF14) per incomplete block, and three replications, at the Embrapa Maize and Sorghum (Sete Lagoas, Minas Gerais, Brazil) experimental station. The low-P soil had ~ 5 ppm of soil P (Mehlich 1) at 0–20 cm depth and between 0 and 5 ppm at the sub-superficial layer (20–40 cm) and the pH was ~ 5.0. At sowing, 200 kg ha^−1^ of NPK formulation 20-00-20 (40 kg ha^−1^ of N, and 40 kg ha^−1^ of K_2_O) were applied to the soil, and 25 days after emergence, the soil was further supplemented with 90 kg ha^−1^ of N, supplied as urea.

### Root morphology in a low-P nutrient solution

Root morphology data was obtained for 272 lines in a paper pouch system with low-P solution as described by de Sousa et al. [56] and Hufnagel et al. [15]. The experiments were set up in a randomized complete block design (RCBD), with three replications. Seeds were sterilized with sodium hypochlorite (0.5%, for five minutes), washed with distilled water, and germinated in paper rolls. After four days, uniform seedlings were selected and transferred to moistened filter paper sheets placed into paper pouches (24 × 33 × 0.020 cm) as described in Hund et al. [57]. Each experimental unit consisted of a pouch with three seedlings, where the bottom 3 cm of each pouch was immersed in containers with 5 L of the nutrient solution whose composition was described by Magnavaca et al. [58] at pH 5.6 and containing 2.5 µM P. The nutrient solution was changed every three days. The containers were kept in a growth chamber for 13 days with a 12 h photoperiod and 27º C and 20º C of day and night temperatures, respectively, under continuous aeration.

After 13 days, the root system was photographed with a digital photography setup, and the images were analyzed with RootReader2D (https://www.quantitative-plant.org/software/rootreader2d) and WinRHIZO (https://regent.qc.ca/assets/winrhizo_software.html) software programs. For multi-trait association mapping, the traits employed were the root morphology traits: root *s*urface *a*rea (SA, cm^2^) and *r*oot *d*iameter (RD, mm), and the performance traits: *s*hoot *d*ry *w*eight (SDW, mg) and *s*hoot *P c*ontent (SPCnt, mg). SPCnt was obtained by multiplying shoot dry weight (g) and shoot P concentration (mg g^−1^), which was analyzed using inductively coupled plasma emission spectrometry [59].

### Root system architecture in a low-P nutrient solution

Root system architecture of 266 sorghum lines was assessed in a hydroponic-based 3D phenotyping system that is a modification of the original RootReader 3D system [15, 19, 60]. The experiments were set up with an augmented block design with two checks (ATF8 and ATF10) per block. Seeds were sterilized and germinated as described above for root morphology assessment. After four days, seedlings were planted between the two top mesh layers of a mesh system using polyethylene foam, created from ABS plastic circles of diameter 20 cm made with a 3D printer (see details in Hufnagel et al. [15]). The mesh systems were placed into clear glass cylinders or in large polyethylene tank containers filled with the nutrient solution described in Magnavaca et al. [58] with 2.5 µM P, maintained at pH 5.6. The containers were kept under continuous aeration in a growth chamber with a 12 h photoperiod and 27º C and 20º C of day and night temperatures, respectively.

Root images for 3D reconstruction of the root system architecture and computation of root architecture traits were taken after ten days with a digital camera. In general, the methods described in detail in Clark et al. [19] were used except that the plants were grown as described above hydroponically instead of in gel cylinders. For each plant’s root system, 100 2D digital images were taken as the plants were rotated 360º with images taken every 3.6º. Then, the image of the plastic mesh system maintaining the root system architecture is digitally removed from the root system image as described in [60] and then the RootReader3D software reconstructs the 3D image of the specific root system from the 100 2D digital images and automatically calculated 19 different root architecture traits as described in Clark et al. [19]. We used for MT-GWAS the 3D traits, *cent*roid (Cent, cm), *med*ian number of *r*oots (MedR) and *c*onvex *h*ull volume (CH, cm^3^).

### Statistical analysis

For grain yield in a low-P soil, the model $${y}_{ijk}=\mu + {R}_{j }+ {B}_{k\left(j\right)}+{Chk}_{{i}^{*}}+{G}_{i\notin {i}^{*}} + {\epsilon }_{ijk}$$ was adopted, where $${y}_{ijk}$$ is the phenotypic value of line $$i$$ in replicate $$j$$ and block $$k$$; $$\mu$$ is the overall mean; $${R}_{j }$$is the fixed effect of $$jth$$ replicate ($$j=1\dots 3)$$, $${B}_{k\left(j\right)}$$ is the random effect of block $$k$$$$(k=1\dots 9, {b}_{k}\sim N\left(0, {\sigma }_{b}^{2}\right))$$ within replicate $$j$$;$${Chk}_{{i}^{*}}$$ is the fixed effect of the check $${i}^{*},{i}^{*}\in C$$, with $$C$$ the set of checks, and $${G}_{i}$$ is the random effect of line $$i(i\notin C,{G}_{i}\sim N\left(0, {\sigma }_{G}^{2}\right)$$. $${\epsilon }_{ijk}$$ is the experimental error assuming $${\epsilon }_{ijk}\sim N\left(0, {\sigma }_{e}^{2}\right)$$.

The statistical model used to analyze the root morphology traits (2D) measured in a low-P nutrient solution was $${y}_{ij}=\mu + {B}_{j }+ {G}_{i}+{\epsilon }_{ij}$$, where $${y}_{ij}$$ is the phenotypic value of line $$i$$ in block $$j$$; $$\mu$$ is the overall mean; $${B}_{j }$$is the fixed effect of block $$j$$ ($$j=1\dots 3)$$; $${G}_{i}$$ is the random effect of line $$i$$$$({G}_{i}\sim N\left(0, {\sigma }_{G}^{2}\right))$$ and $${\epsilon }_{ij}$$ is the experimental error of line $$i$$ in the $$jth$$ block $$({\epsilon }_{ijk}\sim N\left(0, {\sigma }_{e}^{2}\right))$$.

The statistical model adopted to analyze the root system architecture traits (3D) evaluated in nutrient solution with low-P availability was ${y}_{ij}=\mu + {B}_{j }+ {Chk}_{{i}^{*}}+{G}_{i\notin {i}^{*}} +{\epsilon }_{ij}$, where ${y}_{ij}$ is the phenotypic value of line $i$ in the block $j$; $\mu$ is the overall mean; ${B}_{j }$is the random effect of block $j$ (${j=1\dots 14, b}_{j}\sim N\left(0, {\sigma }_{b}^{2}\right))$;${Chk}_{{i}^{*}}$ is the fixed effect of the check ${i}^{*},{i}^{*}\in C$; with $C$ the set of checks, ${G}_{i}$ is the random effect of line $i(i\notin C,{G}_{i}\sim N\left(0, {\sigma }_{G}^{2}\right))$ and ${\epsilon }_{ij}$ is the experimental error of line $i$ in the $jth$ block $({\epsilon }_{ij}\sim N\left(0, {\sigma }_{e}^{2}\right))$. The 3D traits, convex hull and median roots were log-transformed after initial inspection of the residuals.

The effect of sorghum lines in all models was considered random for estimating the genetic variance component $\left({\sigma }_{g}^{2}\right)$ via restricted maximum likelihood (REML) and the heritability coefficients. The line effect was then moved to the fixed part for estimating best linear unbiased estimators (BLUEs), which were used in GWAS. All models were fitted with the GenStat software v.16 [61]. Generalized heritability ${(H}^{2})$ was estimated as proposed by Cullis et al. [62]:

$${H}^{2}=1-\frac{\stackrel{-}{v}BLUP}{{2\sigma }_{g}^{2}}$$

where $\stackrel{-}{v}BLUP$ is the average variance of the differences between two best linear unbiased predictions (BLUPs). Principal component analysis (PCA) was undertaken with adjusted means (BLUES) for the traits, GY, SA, RD, SDW, SPCnt, Cent, MedR and CH. This was performed using R [63] package *FactoMineR* [64].

### Genotypic data

DNA samples of the sorghum lines were genotyped via genotyping-by-sequencing (GBS, Elshire et al. [26]). The DNA fragments (“reads”) were aligned against the sorghum reference genome v.2, and SNP calling was then performed with the TASSEL-GBS pipeline [65] with TASSEL v.5 [66]. Missing genotypic data were imputed using the NPUTE v.4 software [67] and SNPs with a minimum allelic frequency (MAF) inferior to 1% were removed. GWAS was performed with a total of 295,914 SNP markers, which were annotated and had associated functional effects predicted with the SnpEff program [28].

### Multi-trait association mapping

MT-GWAS was performed with a total of 272 lines (traits not scored in a given line were considered as missing data). All traits were standardized to have zero mean and unit total phenotypic variance before MT-GWAS. SNP markers with MAF < 2.5% and missing data exceeding 20% were removed. MT-GWAS was divided into three steps: (1) a null (i.e. without SNPs) multi-trait mixed model was fitted; (2) A chromosome-specific MT-GWAS scan was then performed via a Generalized Least Squares (GLS) approach. (3) Finally, the MT-GWAS model was re-fitted for significant SNPs identified with the GLS scan of step 2 to re-estimate all variance components and obtain SNP effects and significance.

In step 1, the null models lacking SNPs were fitted to estimate the variance-covariance matrix among traits. The statistical model adopted was $${y}_{it}={\mu }_{t}+ {G}_{it}+ {\epsilon }_{it}$$, $${\mu }_{t}$$ is the intercept for each trait; $${G}_{it}$$ is a trait-specific random genotypic effect, assuming $$\sim N\left(0,{\Sigma }\right)$$, with $${\Sigma }={{\Sigma }}_{G}\otimes{{\Sigma }}_{T}$$, where $${{\Sigma }}_{G}$$ is a genetic relationship matrix estimated from SNP markers, $$\otimes$$ denotes the Kronecker product and $${{\Sigma }}_{T}$$ is a variance-covariance matrix between traits. Three structures for the $${{\Sigma }}_{T}$$ matrix were tested, factor analytic order 1 model (FA1 [68, 69], , factor analytic order 2 model (FA2 [70]), and the unstructured model (UNS). The residual followed $${\epsilon }_{it}$$$$\sim N(0,{{\Sigma }}_{R})$$, where $${{\Sigma }}_{R}$$ is a diagonal matrix with trait-specific variances and with no covariance between residuals of different traits. The genetic relationship matrix was obtained following VanRaden [71] and the models were adjusted with *ASReml-R v.3* [72]. The best model was chosen based on the Akaike Information Criterion (AIC, Akaike and Akaikei [73].

In step 2 (single-QTL model), chromosome-specific MT-GWAS via GLS estimation was performed conditional on the variance-covariance matrix obtained on the model with no markers. For this purpose, we re-fitted the model used in step 1, adding an extra effect associated with the SNP markers. The statistical model adopted was ${y}_{it}={\mu }_{t}+{x}_{ir}{\propto }_{rt}+ {G}_{it}+ {\epsilon }_{it},$ where ${x}_{ir}$ represents the genotype of line $i$ for the SNP $r$, assuming the values 0 or 2 for lines that are homozygous for the major and minor alleles, respectively; ${\propto }_{rt}$ is the fixed effect of SNP $r$ on trait $t$; ${G}_{it}$, like in null model, is a trait-specific random genotypic effect, assuming $\sim N\left(0,{\Sigma }\right)$, however with a modified genetic relationship matrix ${{\Sigma }}_{G}^{c} ({\Sigma }={{\Sigma }}_{G}^{c} \otimes {{\Sigma }}_{T})$. The modified genetic relationship matrices were estimated per chromosome in such a way that, for testing SNPs on a given chromosome $c$, the relationship matrix was calculated after excluding all SNPs on that particular chromosome. This approach avoids over correction in GWAS [74] and improves the power for SNP detection. The chromosome-specific GWAS was run in R [63].

The significance threshold for GWAS was based on the Bonferroni correction [75]. The number of independent tests was defined according to an approximate average extent of linkage disequilibrium (LD) of 150 Kb [29], resulting in a *-*log_10_(*p*) threshold equal to 4.94 (*alpha* = 0.05). As the final step (step 3), the full mixed model used in step 2 was re-fitted for markers found to be associated (-log_10_(*p*) ≥ 4.94) and suggestively associated (4 < -log_10_(*p*) < 4.94) in that step to re-estimate all variance components and SNP effects and test their significance with the Wald test [76]. This readjustment is necessary because, different from the GLS model in the full mixed model (step 2), the variance-covariance matrix is now conditional on the fitted SNPs (step 3). MT-GWAS with the full mixed model was performed with *ASReml-R v.3* [72].

The proportion of the phenotypic variance explained by the markers was calculated for all markers, including those with suggestive associations. For that, we use the following formula: $\left(\frac{{V}_{m}}{{V}_{pnull}}\right)x100)$; where ${V}_{m}$ is the marker variance calculated from genotype frequencies in the association panel and the marker effect estimated considering marker as fixed effect (step 3); and ${V}_{pnull}$ is the phenotypic variance of the null model (i.e. without markers, step 1). LD was estimated for all pairs of associated SNPs in the same chromosome. The LD analysis based on the squared genotypic correlations (${r}^{2}$) [30] was extended to include all SNPs up to 2,000 kb downstream and upstream of each associated SNP. The *p*-values associated with the r^2^ estimates were determined by a two-sided Fisher’s Exact test, both performed in R [63].

### RSA, root morphology and P efficiency analysis in soil

For this experiment we selected the genotypes SC25 and SC103, SC1080 and SC413, with SC25 and SC103 showing higher centroids compared to SC1080 and SC413 upon 3D assessment in nutrient solution. These genotypes were cultivated in a PVC cylinder system composed of four cylinders with 25 cm in diameter and a height of 20 cm (see Fig. 6A for details) in a greenhouse with approximately 28ºC and 22ºC of day and night temperatures, respectively, and with irrigation as needed. The topmost cylinder (Ring I in Fig. 6A) was filled with a 0–20 cm layer of a high P fixing Oxisol (Latossolo Vermelho Distrófrico, Santos et al. [31]) with a clayey texture (64% of clay), which is a typical soil found in the Cerrado (i.e. Savanna) biome in in Brazil. Rings II and III were filled with the 20–40 cm and 40–80 cm soil layers, respectively. The bottom-most ring, underneath Ring III, was also filled with soil from the 40–80 cm layer. Prior to filling the PVC cylinders, the soil collected from each of the layers was amended to neutralize Al^3+^, so that the effect of low P on root architecture and morphology can be isolated. Hence, soil from the first layer (0–20 cm) was limed with 6.0 Mg ha^−1^ dolomitic limestone (CaO 42%, MgO 8%, ECCE 80%), recommended based on soil analysis and according to the method of neutralizing Al^3+^ and increasing Ca and Mg contents [77] and 3.50 Mg ha^−1^ of phosphogypsum (Ca 17%, S 14%) was added to supply calcium and sulphur. Five and ten Mg ha^−1^ lime were applied to soil from the 20–40 cm and 40–80 cm layers, respectively. The rings were kept together using adhesive tape, which allowed us to excise the first three rings with a steel cable and assess root morphology and root architecture traits upon harvest. Grain weight (g) data under low P was also acquired. Topsoil fertilization at sowing was undertaken with N-P-K + FTEBR12 (N – 50 kg ha^−1^ as urea, P_2_O_5_ – 200 kg ha^−1^ triple superphosphate [TSP], K_2_O – 240 kg ha^−1^ as KCl and FTEBR12–100 kg ha^−1^). Nitrogen side dressing as urea was applied at 14 and 29 days after planting at a 200 mg N dm^-3^ soil. Irrigation was applied as needed.

Upon harvest, each of the three rings were excised with a steel cable and the roots were cleaned with running water to remove the adherent soil. Root system architecture analysis in the 0–20 cm soil layer (Ring I) was conducted with the Digital Imaging of Root Traits (DIRT, Das et al. [32]), with the modifications described in [78]. Root morphology analysis in all soil layers (Rings I, II and III) was obtained with WinRHIZO and conducted as described in the “Root morphology in a low-P nutrient solution” section above, except that plants were cultivated in a low-P soil. The root architecture traits measured were projected root area (area, cm^2^), median width of root system (cm), and top root angle (degrees). The root morphology traits assessed were root surface area (SA, cm^2^) and root diameter (RD, mm).

The statistical analyses were performed with the R-package *ExpDes.pt* [79]. For grain weight and the root architecture traits, we adopted the model $${y}_{ij}=\mu + {B}_{j }+{G}_{i} +{\epsilon }_{ij}$$, where $${y}_{ij}$$ is the phenotypic value of line $$i$$ in the block $$j$$; $$\mu$$ is the overall mean; $${B}_{j }$$ is the fixed effect of block $$j (j=1\dots 5)$$; $${G}_{i}$$is the fixed effect of line $$i (i=1\dots 4)$$, and $${\epsilon }_{ij}$$ is the experimental error of line $$i$$ in the $$jth$$ block $$({\epsilon }_{ij}\sim N\left(0, {\sigma }_{e}^{2}\right)$$. For the root morphology traits, the treatments consisted of a factorial genotype x soil depth (ring), so the model adopted was an extension of the model previously described, that is, $${y}_{ijk}=\mu + {B}_{j }+{G}_{i}+ {R}_{k}+{GR}_{ik} + {\epsilon }_{ijk}$$, where $${y}_{ijk}$$ is the phenotypic value of line $$i$$ in the block $$j$$ in the $$kth$$ ring, $${R}_{k}$$ is the fixed effect of ring $$k (k=1\dots 3)$$, $${GR}_{ik}$$ is the fixed effect of the line × ring interaction, and $${\epsilon }_{ijk}$$ is the experimental error the line $$i$$, in the block $$j$$ in the ring $$k$$$$({\epsilon }_{ijk}\sim N\left(0, {\sigma }_{e}^{2}\right)$$.

### Supplementary Information

Supplementary Material 1.

Supplementary Material 2.

Supplementary Material 3.

## Data Availability

The datasets generated and/or analyzed during the current study are available in the additional supporting file “Paper_data.zip”. The ASReml-R codes used in this study are available from the corresponding author upon request.

## Abbreviations

AIC: Akaike information criterion
Al: Aluminum
BLUEs: Best linear unbiased estimators
BLUPs: Best linear unbiased predictions
Cent: Centroid
CH: Convex hull
DIRT: Digital imaging of roots traits
DRO1: DEEPER ROOTING 1
FA1: Factor analytic order 1 model
FA2: Factor analytic order 2 model
Fe: Iron
GBS: Genotyping-by-sequencing
GEI: Genotype x environment interaction
GLS: Generalized least squares
GWAS: Genome wide association mapping
GY: Grain yield
LD: Linkage disequilibrium
MAF: Minor allele frequency
MedR: Median number of roots
MT-GWAS: Multi-trait genome wide association mapping
P: Phosphorus
PAE: Phosphorus acquisition efficiency
PC: Principal component
PCA: Principal component analysis
PSTOL1: Phosphorus starvation tolerance 1
QTL: Quantitative trait loci
RD: Root diameter
REML: Restricted maximum likelihood
RIL: Recombinant inbred line
RSA: Root system architecture
RV: Root volume
SA: Root surface area
SAP: Sorghum association panel
SDW: Shoot dry weight
SNP: Single nucleotide polymorphism
SPCnt: Shoot phosphorus content
UNS: Unstructured model
