# Supplementary material for: Multi-trait association mapping for phosphorous efficiency reveals flexible root architectures in sorghum
Source: BMC Plant Biol. 2024 Jun 15;24:562. doi: 10.1186/s12870-024-05183-5 (PMC11179229; doi:10.1186/s12870-024-05183-5)
Supplement: Supplementary file 1 — Supplementary Material 1. [file 12870_2024_5183_MOESM1_ESM.pdf]

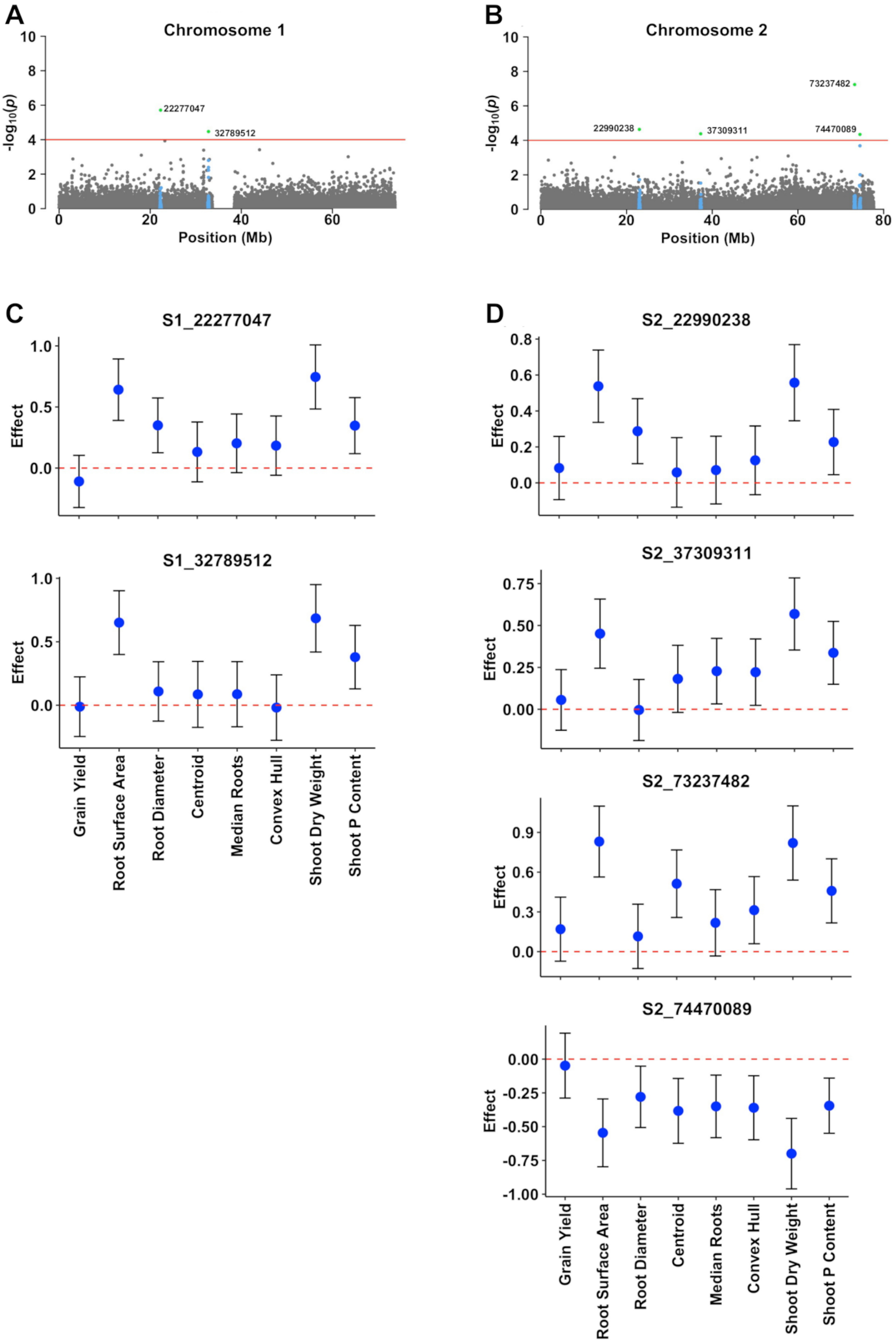

Fig. S1 Estimated effects for SNPs that were significantly associated with multiple traits by MT-GWAS. The physical coordinates (in base pairs, bp, based on the sorghum genome version 2.1), next to each associated SNP are shown for SNPs on chromosomes 1 (**A**) and 2 (**B**). Associated SNPs (green) and SNPs within a physical window of 150 Kb (in blue, depicting the estimated extent of LD in sorghum, Morris et al. [29]) around the associated SNPs are highlighted. The red dashed line depicts the $-\log_{10}(p) = 4$ threshold. Estimated effects for SNPs

on chromosomes 1 and 2 with maximum -$\log_{10}(p)$ by MT-GWAS are shown in (**C**) and (**D**), respectively (explained phenotypic variances for each SNP are Table S3). The SNP designations shown in (**C**) and (**D**) consist of the letter "S" (SNP) followed by the respective chromosome number and physical position in bp. Estimated SNP effects (blue dots) and 95% confidence intervals (vertical line) are shown. The horizontal red dashed line at zero indicates there was no statistically significant difference for the effect of the two homozygous classes at each SNP locus. Hence, the confidence intervals for significant SNPs do not overlap with the dashed line. The effect signs, either positive or negative, indicate the origin of the allele that increases phenotypic expression of a given trait (i.e. favorable allele). SNPs with positive effect signs have the minor allele (allele with a frequency < 0.5) as favorable, whereas negative signs indicate that the alleles with frequency > 0.5 (major allele) increase the phenotype. All traits were standardized before MT-GWAS to have zero means and total phenotypic variance equal to 1.

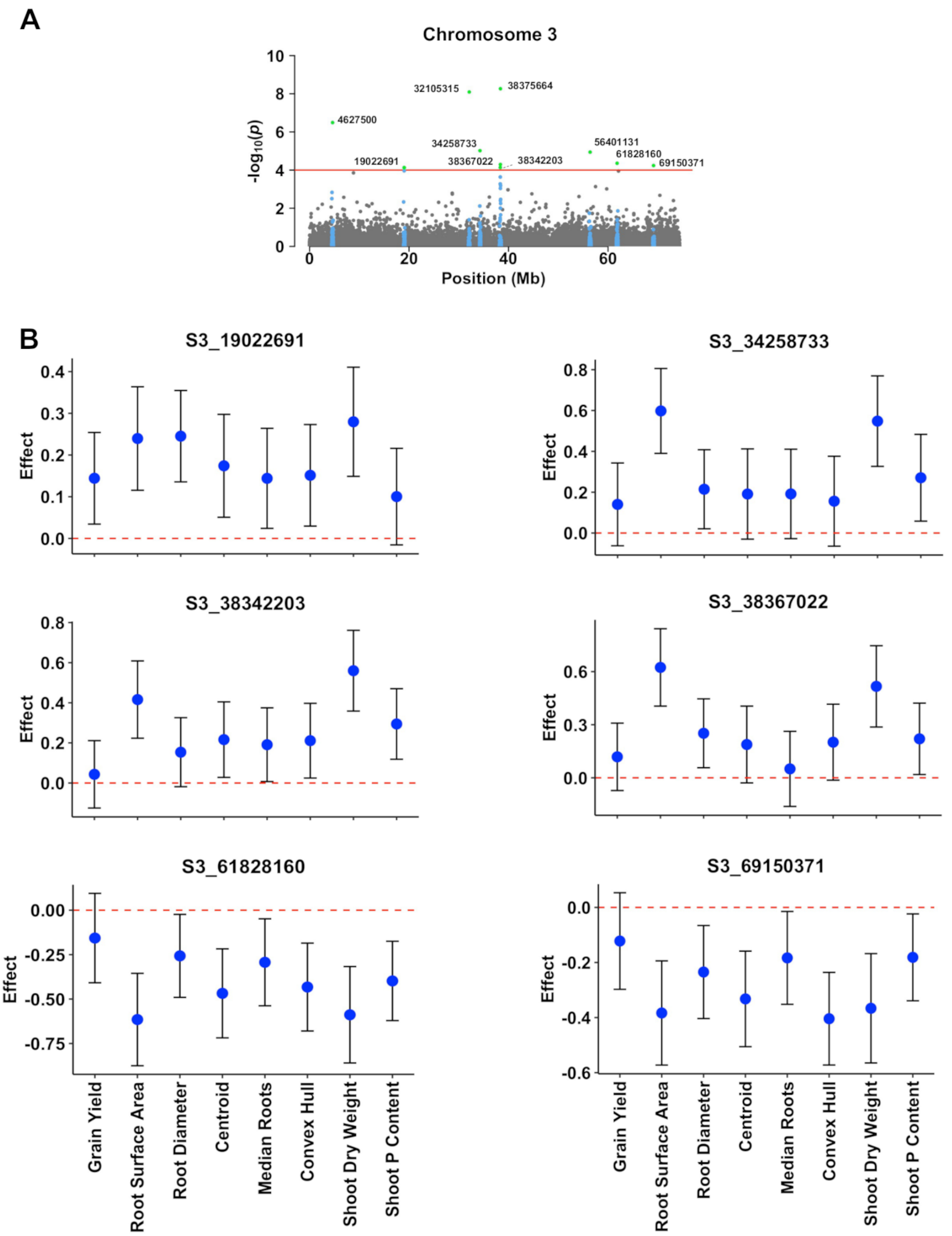

Fig. S2 Estimated effects for additional SNPs that were significantly associated with multiple traits by MT-GWAS. The physical coordinates (in base pairs, bp, based on the sorghum genome version 2.1), next to each associated SNP are shown for SNPs on chromosomes 3 (**A**). Associated SNPs (green) and SNPs within a physical window of 150 Kb (in blue, depicting the estimated extent of LD in sorghum, Morris et al. [29]) around the associated SNPs are highlighted. The red dashed line depicts the $-\log_{10}(p) = 4.0$ threshold. Estimated effects for SNPs on chromosomes 3 with maximum $-\log_{10}(p)$ by MT-GWAS are shown in (**B**) (explained phenotypic variances for each SNP are in Table S3). The SNP designations shown in (**B**) consist of the letter "S" (SNP) followed by the respective chromosome number and physical position

in bp. Estimated SNP effects (blue dots) and 95% confidence intervals (vertical line) are shown. The horizontal red dashed line at zero indicates there was no statistically significant difference for the effect of the two homozygous classes at each SNP locus. Hence, the confidence intervals for significant SNPs do not overlap with the dashed line. The effect signs, either positive or negative, indicate the origin of the allele that increases phenotypic expression of a given trait (i.e. favorable allele). SNPs with positive effect signs have the minor allele (allele with a frequency < 0.5) as favorable, whereas negative signs indicate that the alleles with frequency $>$ 0.5 (major allele) increase the phenotype. All traits were standardized before MT-GWAS to have zero means and total phenotypic variance equal to 1.

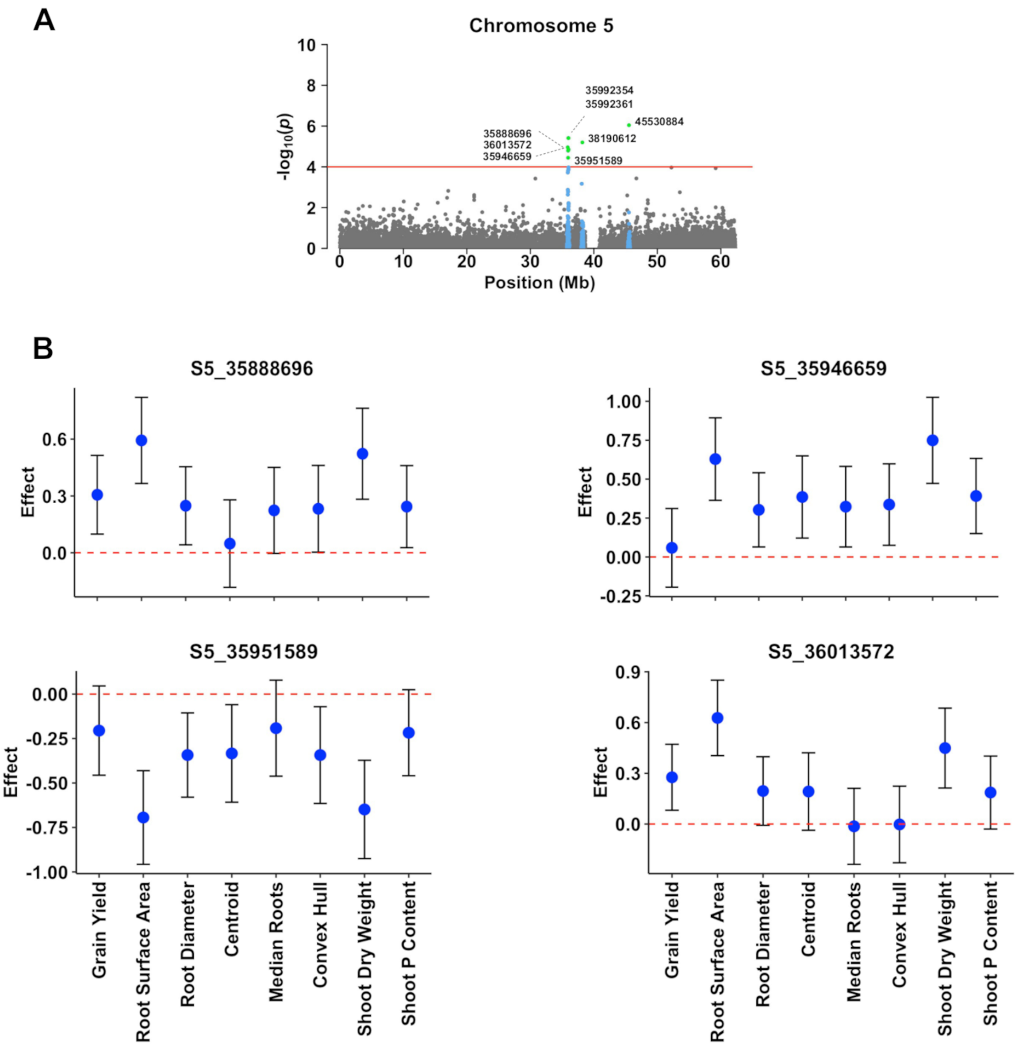

Fig. S3 Estimated effects for additional SNPs that were significantly associated with multiple traits by MT-GWAS. The physical coordinates (in base pairs, bp, based on the sorghum genome version 2.1), next to each associated SNP are shown for SNPs on chromosomes 5 (**A**). Associated SNPs (green) and SNPs within a physical window of 150 Kb (in blue, depicting the estimated extent of LD in sorghum, Morris et al., [29]) around the associated SNPs are highlighted. The red dashed line depicts the -$\log_{10}(p)$ = 4.0 threshold. Estimated effects for SNPs on chromosomes 5 with maximum -$\log_{10}(p)$ by MT-GWAS are shown in (**B**) (explained phenotypic variances for each SNP are in Table S3). The SNP designations shown in (**B**) consist of the letter "S" (SNP) followed by the respective chromosome number and physical position in bp. Estimated SNP effects (blue dots) and 95% confidence intervals (vertical line) are shown. The horizontal red dashed line at zero indicates there was no statistically significant difference for the effect of the two homozygous classes at each SNP locus. Hence, the confidence intervals for significant SNPs do not overlap with the dashed line. The effect signs, either positive or negative, indicate the origin of the allele that increases phenotypic expression of a given trait (i.e. favorable allele). SNPs with positive effect signs have the minor allele (allele with a

frequency < 0.5) as favorable, whereas negative signs indicate that the alleles with frequency > 0.5 (major allele) increase the phenotype. All traits were standardized before MT-GWAS to have zero means and total phenotypic variance equal to 1.

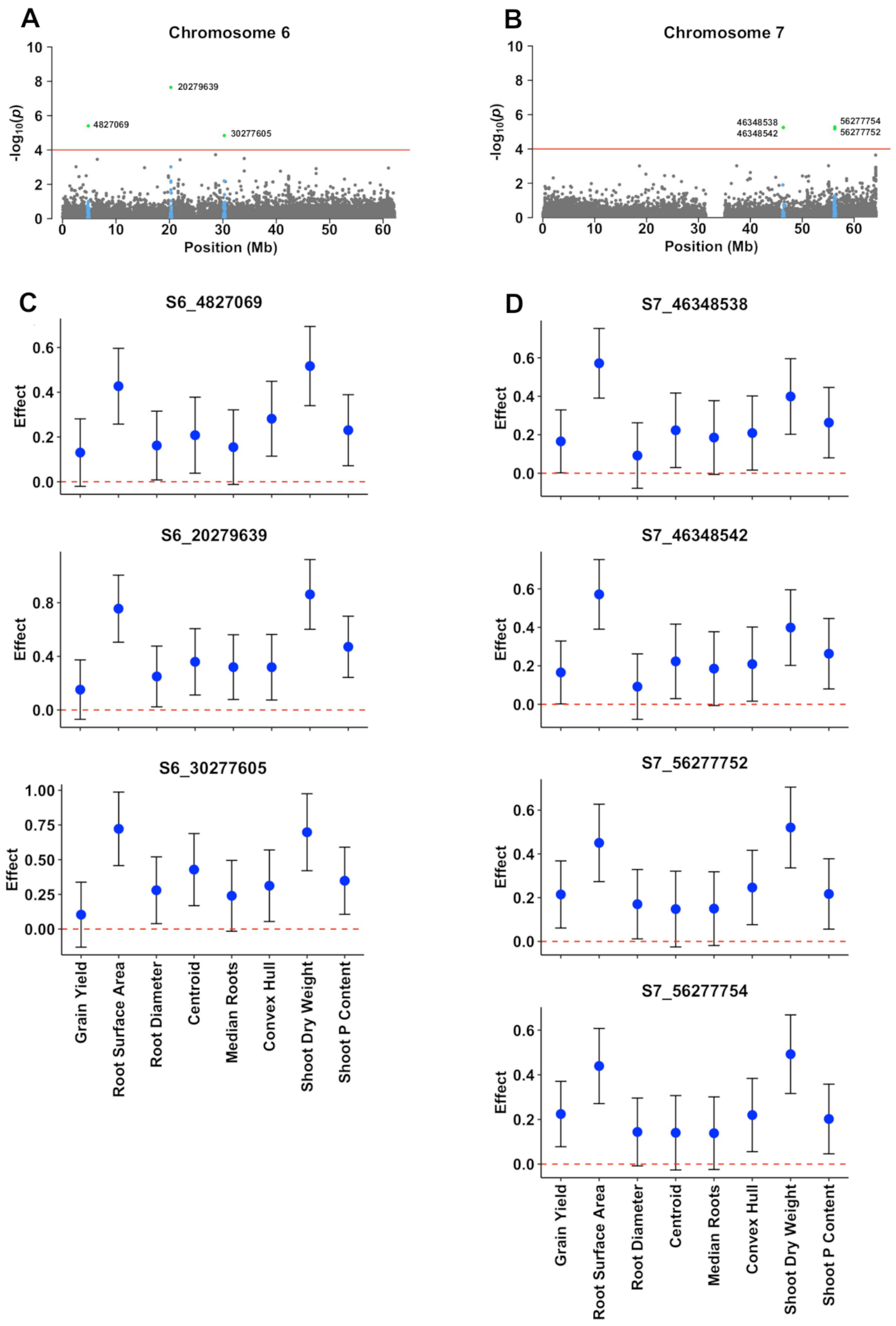

Fig. S4 Estimated effects for SNPs that were significantly associated with multiple traits by MT-GWAS. The physical coordinates (in base pairs, bp, based on the sorghum genome version 2.1), next to each associated SNP are shown for SNPs on chromosomes 6 (**A**) and 7 (**B**). Associated SNPs (green) and SNPs within a physical window of 150 Kb (in blue, depicting the estimated extent of LD in sorghum, Morris et al. [29]) around the associated SNPs are highlighted. The red dashed line depicts the $-\log_{10}(p)$ = 4.0 threshold. Estimated effects for

SNPs on chromosomes 6 and 7 with maximum -$\log_{10}(p)$ by MT-GWAS are shown in (**C**) and (**D**), respectively (explained phenotypic variances for each SNP are in Table S3). The SNP designations shown in (**C**) and (**D**) consist of the letter "S" (SNP) followed by the respective chromosome number and physical position in bp. Estimated SNP effects (blue dots) and 95% confidence intervals (vertical line) are shown. The horizontal red dashed line at zero indicates there was no statistically significant difference for the effect of the two homozygous classes at each SNP locus. Hence, the confidence intervals for significant SNPs do not overlap with the dashed line. The effect signs, either positive or negative, indicate the origin of the allele that increases phenotypic expression of a given trait (i.e. favorable allele). SNPs with positive effect signs have the minor allele (allele with a frequency < 0.5) as favorable, whereas negative signs indicate that the alleles with frequency > 0.5 (major allele) increase the phenotype. All traits were standardized before MT-GWAS to have zero means and total phenotypic variance equal to 1.

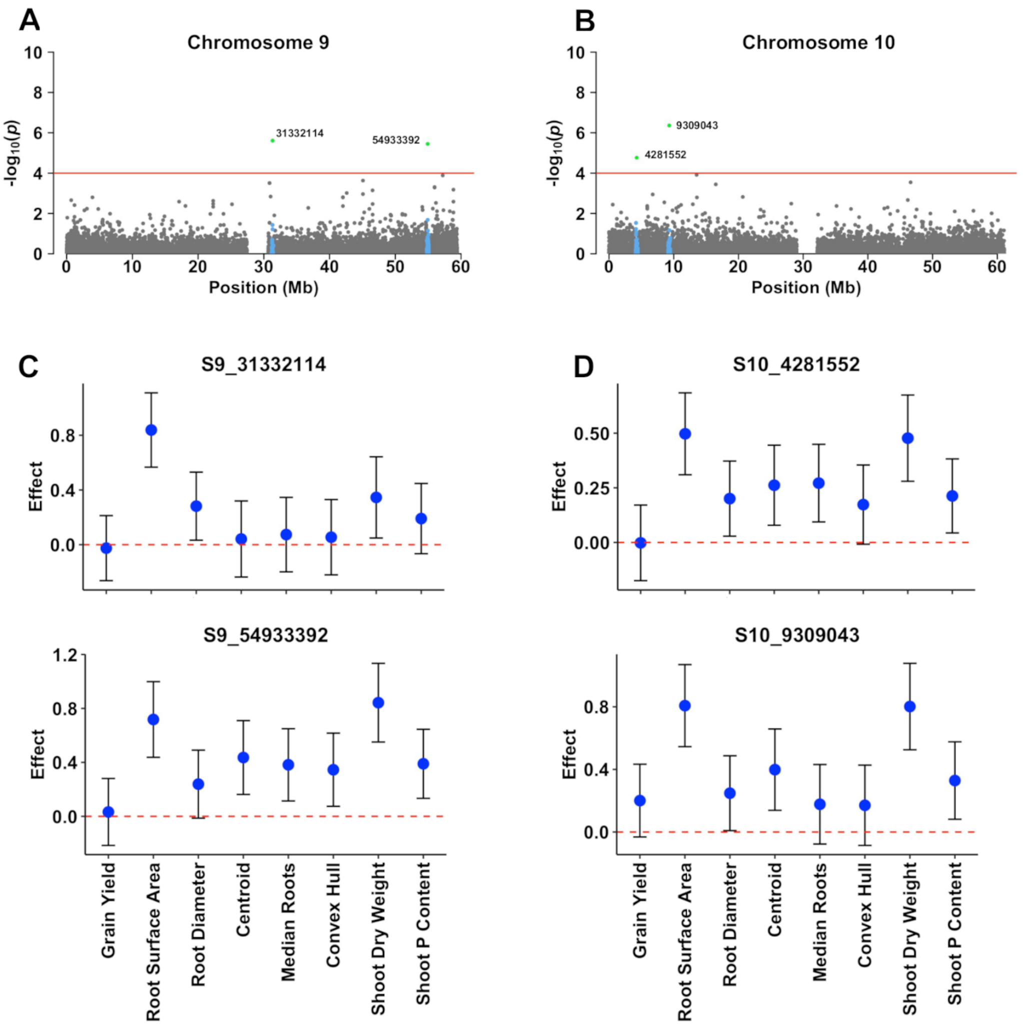

Fig. S5 Estimated effects for SNPs that were significantly associated with multiple traits by MT-GWAS. The physical coordinates (in base pairs, bp, based on the sorghum genome version 2.1), next to each associated SNP are shown for SNPs on chromosomes 9 (**A**) and 10 (**B**). Associated SNPs (green) and SNPs within a physical window of 150 Kb (in blue, depicting the estimated extent of LD in sorghum, Morris et al., [29]) around the associated SNPs are highlighted. The red dashed line depicts the $-\log_{10}(p)$ = 4.0 threshold. Estimated effects for SNPs on chromosomes 9 and 10 with maximum $-\log_{10}(p)$ by MT-GWAS are shown in (**C**) and (**D**), respectively (explained phenotypic variances for each SNP are in Table S3). The SNP designations shown in (**C**) and (**D**) consist of the letter "S" (SNP) followed by the respective chromosome number and physical position in bp. Estimated SNP effects (blue dots) and 95% confidence intervals (vertical line) are shown. The horizontal red dashed line at zero indicates there was no statistically significant difference for the effect of the two homozygous classes at each SNP locus. Hence, the confidence intervals for significant SNPs do not overlap with the dashed line. The effect signs, either positive or negative, indicate the origin of the allele that

increases phenotypic expression of a given trait (i.e. favorable allele). SNPs with positive effect signs have the minor allele (allele with a frequency < 0.5) as favorable, whereas negative signs indicate that the alleles with frequency > 0.5 (major allele) increase the phenotype. All traits were standardized before MT-GWAS to have zero means and total phenotypic variance equal to 1.
